# Biogeochemical Niche of Magnetotactic Cocci Capable of Sequestering Large Polyphosphate Inclusions in the Anoxic Layer of the Lake Pavin Water Column

**DOI:** 10.3389/fmicb.2021.789134

**Published:** 2022-01-10

**Authors:** Cécile C. Bidaud, Caroline L. Monteil, Nicolas Menguy, Vincent Busigny, Didier Jézéquel, Éric Viollier, Cynthia Travert, Fériel Skouri-Panet, Karim Benzerara, Christopher T. Lefevre, Élodie Duprat

**Affiliations:** ^1^Sorbonne Université, Muséum National d’Histoire Naturelle, UMR CNRS 7590 – Institut de Minéralogie, de Physique des Matériaux et de Cosmochimie (IMPMC), Paris, France; ^2^Aix-Marseille University, CNRS, CEA, UMR 7265 Institute of Biosciences and Biotechnologies of Aix-Marseille, CEA Cadarache, Saint-Paul-lez-Durance, France; ^3^Université de Paris, Centre de Recherches Interdisciplinaires (CRI), Paris, France; ^4^Université de Paris, Institut de Physique du Globe de Paris, CNRS, Paris, France; ^5^INRAE & Université Savoie Mont Blanc, UMR CARRTEL, Thonon-les-Bains, France; ^6^LSCE, CEA/CNRS/UVSQ/IPSL, Université Paris Saclay & Université de Paris France, Gif-sur-Yvette Cedex, France

**Keywords:** magnetotactic bacteria (MTB), magnetosomes, redox and chemical gradients, morphotype diversity, P sequestration, electron microscopy, intracellular inclusions, polyphosphates (PolyP)

## Abstract

Magnetotactic bacteria (MTB) are microorganisms thriving mostly at oxic–anoxic boundaries of aquatic habitats. MTB are efficient in biomineralising or sequestering diverse elements intracellularly, which makes them potentially important actors in biogeochemical cycles. Lake Pavin is a unique aqueous system populated by a wide diversity of MTB with two communities harbouring the capability to sequester not only iron under the form of magnetosomes but also phosphorus and magnesium under the form of polyphosphates, or calcium carbonates, respectively. MTB thrive in the water column of Lake Pavin over a few metres along strong redox and chemical gradients representing a series of different microenvironments. In this study, we investigate the relative abundance and the vertical stratification of the diverse populations of MTB in relation to environmental parameters, by using a new method coupling a precise sampling for geochemical analyses, MTB morphotype description, and *in situ* measurement of the physicochemical parameters. We assess the ultrastructure of MTB as a function of depth using light and electron microscopy. We evidence the biogeochemical niche of magnetotactic cocci, capable of sequestering large PolyP inclusions below the oxic–anoxic transition zone. Our results suggest a tight link between the S and P metabolisms of these bacteria and pave the way to better understand the implication of MTB for the P cycle in stratified environmental conditions.

## Introduction

Magnetotactic bacteria (MTB) refer to a unique group of morphologically, phylogenetically, and physiologically diverse microorganisms thriving mostly at oxic–anoxic boundaries of aquatic habitats. They form intracellular magnetic nanocrystals enclosed within vesicles named magnetosomes that vary between species in size, shape, composition (Fe_3_O_4_ or Fe_3_S_4_), number, and organisation (Schüler, 1999; Isambert et al., 2007). The magnetic properties of these magnetosomes allow MTB cells to passively align parallel to the geomagnetic field lines and swim actively towards, or away, attractants and repellents, in chemically stratified environments (Lefevre and Bazylinski, 2013). Most known MTB species are microaerophiles or anaerobes and were essentially described at water-sediment interfaces (Lefevre and Bazylinski, 2013). Because of their ability to trigger the formation of magnetic biominerals into unique prokaryotic organelles and their potential contribution to the Fe biogeochemical cycle (Chen et al., 2014; Lin et al., 2014; Amor et al., 2020), MTB have been for decades the focus of numerous studies in many fields of research, from microbial ecology, evolution (Lin et al., 2018; Monteil et al., 2018), molecular genetics (Uebe and Schüler, 2016), palaeomagnetism (Kobayashi et al., 2006; Li et al., 2013) to geochemistry (Amor et al., 2015) and biophysics (Popp et al., 2014; Keim et al., 2018; Klumpp et al., 2019).

Recent studies of MTB in natural environments revealed additional underestimated biomineralisation processes in this group of bacteria, providing a potential broad impact of MTB on P, S, and C geochemical cycles (Li et al., 2021). In particular, MTB observed at the oxic–anoxic transition zone (OATZ) in the stratified water column and in sediments of the ferruginous Lake Pavin (Massif Central, France) harbour the capability to sequester phosphorus and cations (Ca^2+^, Mg^2+^, or K^+^) in the form of very large inclusions of polyphosphates (PolyP), or carbon and calcium in the form of amorphous calcium carbonates (Rivas-Lamelo et al., 2017; Monteil et al., 2021). Previous studies showed that phosphogenesis in this lake occurs just beneath the OATZ (Busigny et al., 2016) and suggested the involvement of cells in mineral precipitations (Cosmidis et al., 2014; Miot et al., 2016). In this freshwater environment, magnetotactic cocci (MTBc) hyperaccumulating P (up to 90% of their cell volume) as intracellular PolyP were affiliated to the *Magnetococcaceae* family of the *Candidatus* Betaproteobacteria class—also proposed as the class Magnetococcia in the GTDB taxonomy (Ji et al., 2017; Parks et al., 2018). Based on the 16S rRNA gene sequence, two different species of MTBc were detected (Rivas-Lamelo et al., 2017). MTB forming 2–4 large granules of intracellular amorphous CaCO_3_ (iACC), occupying up to 65% of their cell volume, were affiliated to two undescribed genera within the *Rhodospirillaceae* family of the Alphaproteobacteria (Monteil et al., 2021). Intracellular carbonatogenesis coupled with magnetosome formation in magnetotactic *Alphaproteobacterium* was also recently evidenced in sediment of Xingqinggong Lake (Liu et al., 2021). Before these observations, only Cyanobacteria and the non-magnetotactic Gammaproteobacterium *Achromatium* were known to form carbonate phases in undersaturated solutions (Head et al., 1996; Couradeau et al., 2012; Salman et al., 2015; Cam et al., 2016; Benzerara et al., 2020).

Polyphosphate inclusions have been observed within cells of many bacteria, microalgae, diatoms, fungi, yeasts, plants, and animals (Kulaev et al., 2004; Achbergerová and Nahálka, 2011). PolyP are linear polymers of orthophosphate linked together by high-energy phosphoanhydride bonds (Kornberg et al., 1999; Achbergerová and Nahálka, 2011), whose energy is released and used for biological reactions upon breaking (Kulaev, 1975). The PolyP storage usage for microorganisms is multiple, among which are energy storage (Bonting et al., 1991; Kornberg, 1995), motility (Seufferheld et al., 2008; Varela et al., 2010; Möller et al., 2019), chaperone activity (Kampinga, 2014), chelator for metal ions (Kornberg, 1995), virulence, or even antibiotic resistance (Wang et al., 2018; Bowlin and Gray, 2021). Alternatively, Lechaire et al. (2002) hypothesised that PolyP accumulation may serve for oxygen storage. The current paradigm suggests that few bacterial genera, e.g., the marine sulphur oxidisers *Thiomargarita* and *Beggiatoa*, are particularly efficient in the sequestration of P and accumulation as PolyP under oxic conditions and hydrolyse them back into orthophosphates under anoxic/sulphide-rich conditions (Mußmann et al., 2007; Brock and Schulz-Vogt, 2011). This process appears to be involved in phosphogenesis at modern water–sediment interfaces in upwelling zones (e.g., continental margin of Peru or Namibia) (Schulz and Schulz, 2005; Crosby and Bailey, 2012; Mänd et al., 2018). Moreover, few genera of PolyP-accumulating microorganisms such as *Accumulibacter* sp. have been described as key components for P removal in wastewater treatment plants, enriched through anaerobic/aerobic cycling inducing P sequestration at the interface between sludge and sewage sludge (Kawakoshi et al., 2012; Kristiansen et al., 2013; Oyserman et al., 2016). However, recent discoveries of bacteria harbouring large amounts of intracellular PolyP under constant anoxia challenge this paradigm. This is the case for bacteria affiliated to the *Sulfurimonas* genus in the water column of the Baltic Sea (Möller et al., 2019) and MTB affiliated to the *Magnetococcaceae* family from the water column of both Lake Pavin and the Black Sea (Rivas-Lamelo et al., 2017; Schulz-Vogt et al., 2019). Although PolyP inclusions were frequently described in MTB cells (Lins and Farina, 1999; Bazylinski et al., 2004, 2013; Oestreicher et al., 2011; Lefèvre et al., 2012), the capability of P hyperaccumulation seems yet to be specific of MTB affiliated to the *Magnetococcaceae* family (Cox et al., 2002; Eder et al., 2014; Chen et al., 2015; Schulz-Vogt et al., 2019; Li et al., 2020b). Both diversity and unicity of biological and environmental parameters governing these processes of sequestration of large amount of P remain to be better understood.

The genetic and metabolic bases of PolyP and CaCO_3_ hyperaccumulation in different MTB groups remain unknown. In each group, the sequestration of these elements may be the response to their stratification according to specific environmental conditions that remain to be determined. MTBc with and without PolyP were both observed by Rivas-Lamelo et al. (2017) in the water column of Lake Pavin. The factors related to this cellular heterogeneity remain unknown. In the absence of cultivated models, an *in situ* characterisation of the geochemical and physicochemical parameters structuring populations and their bioremediation capabilities can directly or indirectly provide answers, and to a larger extent, it may help in designing a cultivation protocol. Among known MTB habitats, the OATZ that stretch over a few to several metres in some stratified water columns are particularly suitable to conduct such investigations, in comparison with OATZ in aquatic sediments which are usually restricted to a few millimetres in thickness. The permanently stratified water column of Lake Pavin therefore represents a model site (Sime-Ngando et al., 2016), showing oxygen, chemical, and redox gradients extending over a few metres in depth (Michard et al., 1994; Sime-Ngando et al., 2016).

In the present work, we investigated the depth stratification of the diverse MTB populations in relation to environmental parameters in the water column of Lake Pavin. Using a recently developed methodology coupling *in situ* measurements and water sampling with a high vertical resolution (Busigny et al., 2021), we localised the depth range in the OATZ where the MTB community thrive. We collected water samples from a transect at the redox boundary with a 20-cm interval and examined the MTB morphotype diversity and abundance using magnetic sorting and light microscopy approaches. A classification schema of MTBc is proposed, based on the ultrastructural heterogeneity of PolyP inclusions and magnetosome organisation using transmission electron microscopy. Statistical relationships between the abundance of each MTB morphotype and different physicochemical and geochemical parameters were explored. We identified the biogeochemical niches of MTB populations, in particular PolyP-forming MTBc, and we suggested some key factors associated with metabolisms that could lead to their structuration in natural environments.

## Materials and Methods

### Site Description and Water Sampling Strategy

**FIGURE 1:**
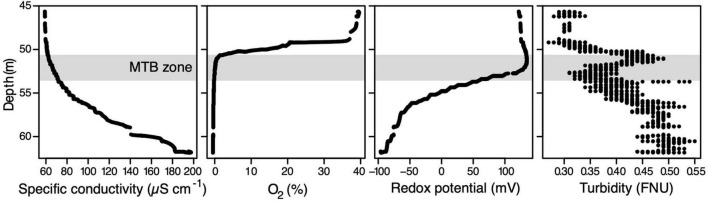
Physicochemical depth profiles in the water column of Lake Pavin measured *in situ* on October 30th. The light grey zone corresponds to the MTB zone, i.e., the water layer inhabited by MTB. FNU, formazin nephelometric unit.

The water column of the ferruginous meromictic Lake Pavin, France (45.495792°N, 2.888117°E), was sampled in October 2019 from a platform anchored at its centre where the lake is the deepest (∼90 m). Previous campaigns showed that MTB are found under the OATZ at depths where dioxygen becomes undetectable and in a specific range of conductivity (Busigny et al., 2021). The vertical localisation of this zone varied slightly over the seasons due to lake dynamics. Therefore, a first physicochemical profile (including dissolved O_2_ concentration and conductivity) of the water column between 45 and 62 m was acquired in October 29, 2019 (1st day), to pinpoint the depth of OATZ and the MTB communities with a 50-cm resolution and was checked again the following morning. No significant change was observed between these two profiles. Then, a fine sampling strategy was established along the few metres from above to below the OATZ on the October 30th afternoon (2nd day) (Figure 1). When reaching 50.7 m (i.e., depth close to the one of first MTB appearance on the 1st day), 9 samples vertically spaced by ∼20 cm on average were collected and further used to determine the MTB cell concentration, diversity, and physicochemical features. Samples were collected using a flexible metered hose following the same protocol as that of Busigny et al. (2021). The ∼65-m-long hose was connected at its near-surface end to a speed-adjustable 12-V brushless pump (DC50E-1250A). For all sampled depths, the hose was purged for ∼5 min (∼2 times the dead volume) before collection of the water. Several samples were collected at each depth for either chemical or biological analysis. For MTB population analysis using light and electron microscopy, samples were collected in several 1-l glass bottles filled to their capacity and tightly closed. At each sampled depth of the water column, 3 l of the sample was filtrated online under anoxia (N_2_ influx and control of oxygen concentration in the gas contained in the filtration device with a multiparameter portable metre) through 47-mm quartz filters with a *ca.* 0.7-μm nominal threshold for chemical analyses. A triplicate of 10 ml soluble fraction was recovered and then acidified with 3 to 4 drops of 65% HNO_3_ Suprapur to prevent any subsequent precipitation. In parallel, the particulate fraction was dried by continuous N_2_ influx in the filtration system after the water entry stopped. The filters were then stored in Petri dishes until further treatments.

### *In situ* Measurements of Physicochemical Parameters

Some physicochemical parameters were measured *in situ* using an YSI EXO2 CTD multiparameter probe, such as the conductivity, temperature, pH, oxidoreduction potential (ORP), dissolved oxygen concentration, turbidity, fluorescent dissolved organic matter (fDOM) concentration, and chlorophyll concentration. The detection limit for dissolved O_2_ is *ca* 0.1% or about 0.3 ± 0.2 μM. Turbidity was also measured simultaneously *in situ* with an Aquatec AQUAlogger 210TYPT (Seapoint turbidity sensor, 880 nm) probe to get a lower sensitivity threshold. An oxygen sensor was calibrated against water vapour-saturated ambient air (100% O_2_ local saturation), and the “zero” O_2_ saturation was checked using a 10% wt. sodium sulphite solution. Specific conductivity at 25°C was calibrated against a KCl standard solution (1,413 μS cm^–1^) and pH using 3 buffers (4/7/10). The different *in situ* probes were fixed at the exact extremity of the sampling hose.

### Estimation of Magnetotactic Bacteria Population Sizes and Analysis of Morphotype Diversity

Populations of north-seeking MTB were quantified by retrieving 40 μl of water sample, and cells were counted using the hanging drop technique (Schüler, 2002) with a ZEISS Primo Star light microscope equipped with contrast and differential interference contrast optics. South-seeking MTB were also counted but were scarce, sometimes below the detection limit. The cell concentration of MTB at each depth of the water column was estimated based on two biological replicates. For each of the 9 samples collected at or around the OATZ, the diversity of MTB morphotypes was also estimated. As previously done (Rivas-Lamelo et al., 2017; Monteil et al., 2021), each counted cell (∼5 to 130 cells per sample) was classified according to its shape (e.g., spirillum, coccoid, rod shape), its size, and the presence/absence of bright inclusions.

Magnetotactic bacteria can also be differentiated by their magnetosome shape, composition, number, and organisation. These characteristics are not observable by light microscopy; thus, the morphotype heterogeneity of the MTB was further analysed using scanning transmission electron microscopy (STEM). MTB were concentrated using a magnetic enrichment protocol that consists in placing the south pole of a stirring magnet against the side of a 1-l bottle at its centre for 1 h. These bottles for each depth correspond to a duplicate of the ones used for light microscopy. Fifteen microlitres of the MTB pellet was deposited onto Cu–carbon TEM grids coated with poly-L-lysine, for 20 min with a stirring magnet below with the south pole facing up (i.e., to attract north-seeking MTB). The grids were then washed twice with filtered sterilised water, and the excess was absorbed after 5 min with a Whatman paper until complete desiccation. STEM observations were performed in the high-angle annular dark-field (HAADF) mode using a JEOL-2100F, operating at 200 kV, and equipped with a JEOL XEDS detector.

### Chemical Analyses of Dissolved and Particulate Fractions in the Water Column

For the measurement of the concentration of major and minor elements (Al, B, Ba, Ca, Fe, K, Li, Mg, Mn, Na, P, S, Si, Sr, Ti) in the particulate fraction using inductively coupled plasma-optical emission spectroscopy (ICP-OES), a 17-mm disc was sliced from each 47-mm quartz membrane and submitted to an acidified solution of 1 M HNO_3_–Suprapur for a period of at least 1 day. Additional concentrations due to a potential release from quartz membranes were shown to be negligible. The subsequent solution was then recovered and diluted in order to fit the range of accurate measurements on the spectrometer (ICP-OES iCAP 6200, Thermo Scientific, Waltham, MA, United States). Considering the analytical error for ICP-OES and from the filtration protocol, a maximum error of ±7.5% was inferred. The ICP-OES was also used to measure the concentrations of chemical elements in the dissolved fraction collected and acidified directly on the field. The sulphide species were not determined due to an unavoidably too large delay between the sampling and the analyses leading to reoxidation of the reduced chemical species. Triplicates of 9-mm sliced discs from the same 47-mm quartz filters used for ICP-OES were recovered for the elemental quantification of particulate organic carbon and nitrogen after carbonate removal using orthophosphoric acid. These quantifications were carried out using an Organic Elemental Analyser CHNS Flash 2000 (Thermo Scientific). Considering classical variability between 2 different CHNS analyses issued from the same 47-mm filter, a maximum error of ± 5% was inferred. For each water sample, a part of the filtrated solution was supplemented with zinc acetate in order to precipitate the potential trace concentration of ΣH_2_S and used for the measurement of sulphate and other anions (fluoride, chloride, bromide, nitrate) by ion chromatography with a Dionex Chromatograph (conventionally accepted error of ±5%). This protocol avoids some artefacts on SO_4_^2–^ determination due to reoxidation of sulphides if they are present in the water.

### Definition and Relative Quantification of Categories of Magnetotactic Cocci Cells According to Their Magnetosome Organisation and the Size of Their Polyphosphates Inclusions

Magnetotactic cocci were differentiated from other MTB cells based on elemental maps of P and Ca, determined by STEM and X-ray energy-dispersive spectroscopy (XEDS). The elemental maps of Fe and O were used to confirm that the MTBc magnetosomes corresponded to magnetite as shown by Rivas-Lamelo et al. (2017). The TEM grid preparation was described above (see section “Materials and Methods: Estimation of Magnetotactic Bacteria Population Sizes and Analysis of Morphotype Diversity”). Each TEM grid was first observed at a very low magnification to evaluate the bacterial abundance on the whole grid and to check their distribution homogeneity. Cells were counted with replicates corresponding to randomly chosen grid squares (80 μm in edge size) when the number of cells was higher than twenty per replicate. Otherwise, all the cells deposited on the grid were counted, without replicate. For each of the 9 sampled depths, the relative proportions of the different categories of MTBc with various PolyP inclusion sizes and magnetosome organisations were quantified on each replicate.

### Statistical Analyses

Statistical analyses of the environmental and biological datasets were performed using the R software, version 4.0.0 (R Core Team, 2020). Our dataset describing the environmental context where MTB have been detected in October 2019 consisted of 8 physicochemical variables (*in situ* measurements) and 37 geochemical variables (concentrations measured by ICP-OES, CHNS Elemental Analyser, or ion chromatography), with observations corresponding to 9 water column depths. Geochemical variables with values under the detection limits for all the 9 depths were excluded (i.e., the concentrations of particulate fractions for lithium, boron, and silicon; concentrations of dissolved fractions for aluminium and lithium; concentrations of bromide in the dissolved fraction). Our final geochemical dataset consisted of a total of 31 variables. Some values were missing in the physicochemical dataset, which we addressed by using modelling strategies. Indeed, because of a failure of the real-time recording device, 1–3 out of 9 values could not be recorded for the following *in situ* measurements: conductivity, temperature, pH, redox potential (ORP), turbidity, chlorophyll concentration, and fDOM. For each of these variables, we built linear regression models using the 6–8 values recorded on the 30th in the afternoon and those corresponding to the same depths in the complete profile realised on the 30th in the morning. Missing values were replaced by values predicted by the model if the linearity was significant (Pearson’s test *p*-value < 0.05) when all test assumptions were satisfied (i.e., independency of residuals, normal distribution of residuals, homogeneous distribution). If not, the variable was excluded from the whole analysis. This approach was successful for the four following variables only: conductivity, temperature, pH, and fDOM concentrations. The other variables—redox potential (ORP), turbidity, and chlorophyll concentration—were excluded from the final environmental dataset which consisted of a total of 35 variables.

First, the strength and direction of the pairwise linear or monotonic relationships between the 35 environmental variables on 9 water column depth samples were evaluated by Pearson’s (when bivariate normality assumptions were satisfied) or Spearman’s correlation coefficients, respectively (values ranging from –1 to +1), using the package *stats* (R Core Team, 2020) and *Hmisc* (Harrell, 2020). The statistical significance of each correlation was assessed and indicated by a *p*-value: correlations were considered as significant when the *p*-value was lower than 8.4 × 10^–5^ (Bonferroni correction corresponding to a false positive error rate lower than 5% per test). Pairwise relationships of the variables were drawn using the R package *corrplot* (Wei and Simko, 2017).

In order to define environmental niches in the water column where MTB populations were observed, an additional clustering of the environmental variables into directional groups associated with a latent variable was performed using the R package *ClustVarLV* (Vigneau et al., 2015). Based on the variation of the clustering criterion after consolidation of the partitions by means of the partitioning algorithm, an important loss in homogeneity of the clusters with 9 clusters was observed which means that a partition into 10 clusters of variables should be retained (Supplementary Figure 1). We performed a standardised principal component analysis (PCA) on the 9 water column depths described by these latent variables (potentially indicative of niche structuration) using the R package *FactoMineR* (Lê et al., 2008).

Our biological dataset describing the distribution and composition of the MTB communities from 9 depths in the water column was composed of variables related to the total abundance of MTB cells and the abundance of each two main MTB populations (MTBc and iACC-forming rods), as determined by light microscopy (Supplementary Table 1). The strength and direction of the pairwise linear or monotonic relationships between the biological and environmental variables were evaluated by Pearson’s (when bivariate normality assumptions were satisfied) or Spearman’s correlation coefficient, respectively. Correlations were considered as significant when the *p*-value was lower than 4.8 × 10^–4^ (Bonferroni correction corresponding to a false positive error rate lower than 5% per test).

Based on our TEM-based classification schema with replicates at four water column depths, the following biological features were also accounted: (i) relative proportions of MTBc without PolyP, with small or large PolyP, (ii) relative proportions of MTBc with two single chains or disorganised magnetosomes, and (iii) the proportion of MTBc with or without PolyP, for each type of magnetosome organisation. The average relative proportions were compared between pairs of samples with replicates by a non-parametric Mann–Whitney–Wilcoxon test. Differences were considered as significant when *p*-values were lower than 0.05.

## Results

### Magnetotactic Bacteria Communities Thrive Below the Oxic–Anoxic Transition Zone in a Zone Vertically Structured in Discrete Geochemical Niches

The physicochemical profile acquired on the first day in Lake Pavin allowed us to localise the OATZ and MTB communities in the water column (Figure 1). In detail, the oxygen concentration of the water column underwent a steep decrease around the 49.7-m depth from ∼40% down to the probe detection limit and complete anoxia at around 50.3 m. In line with previous observations (Busigny et al., 2021), specific conductivity, dissolved organic matter concentration, and temperature gradually increased with depth from ∼60 to >200 μS cm^–1^, from 1 to 5 relative fluorescence units, and from 4°C to >4.5°C, respectively. Moreover, a typical small turbidity peak was observed at a depth just below oxygen disappearance. A rough estimation of the MTB population sizes confirmed their localisation in the anoxic part of the water column between 51.3 and 53.5 m.

**FIGURE 2:**
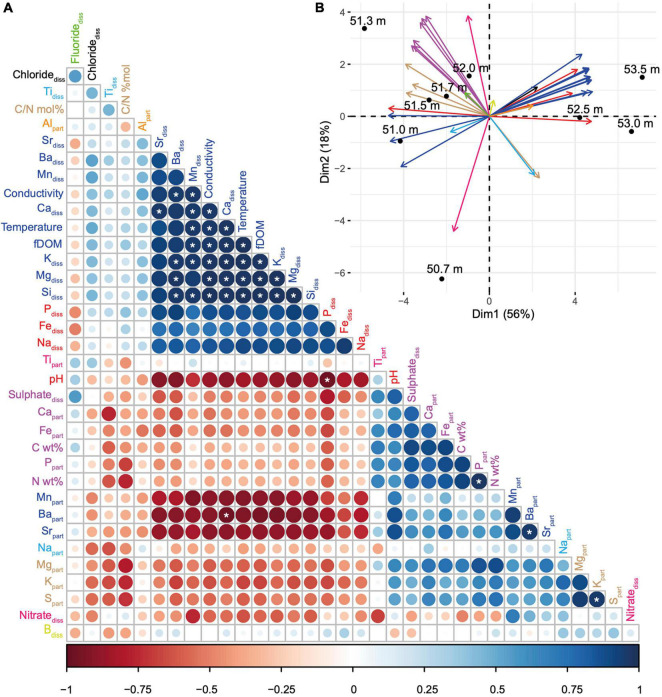
Correlations between the environmental depth profiles and definition of vertical geochemical niches in the MTB zone. **(A)** Correlation matrix built from 35 environmental variables, reordered according to the Pearson’s correlation coefficient. The size and colour of the circles are related to the correlation coefficient value: the bigger and the darker the circle, the better the correlation. Blue and red circles refer to positive and negative correlations, respectively. The white stars indicate significant linear correlations (Pearson’s test). Variables with the same colour belong to the same group as defined by the clustering of variables around latent variables (see Supplementary Figure 1 for details). The conductivity corresponds to the specific conductivity (μS cm^–1^). **(B)** Principal component analysis with biplot of the 35 environmental variables (vectors represented by arrows, using the same colour code than in panel **A**) and the 9 sampled depths (dots). Each principal component (or axis) is a linear combination of variables defined in order to maximize the discrimination of the sample depths; coordinates of vectors on each axis indicate the coefficient of the variables in the linear combination. The smaller the angle between arrows, the stronger the positive linear correlation between the corresponding variables. Orthogonal arrows indicate no correlation, while arrows pointing in opposite directions indicate a negative correlation.

Based on these preliminary data, we performed an additional sampling extending over a few metres in depth specifically where MTB were observed but with a higher depth resolution. At 50.7 m and below, a total of 9 samples spaced by ∼20 cm on average were collected and further used to identify geochemical niches in relation to MTB cell concentration and diversity. By analysing a total of 35 environmental variables at each depth, we identified groups of correlated variables, i.e., with similar depth variations (Figure 2A). Two main groups of geochemical variables can be observed that are negatively correlated with each other. One group mainly corresponds to the dissolved elements, whereas the other group corresponds to the elements in the particulate fraction. Dissolved sulphate appears as one exception since it is strongly positively correlated with particulate Ca, Fe, and P and particulate organic C and N. The two first axes of the principal component analysis built from this matrix explained 74% of the total variance and showed that depths could be divided in three global niches: one at 50.7 m (1 sample), a second from 51 to 52 m (5 samples), and a third one from 52.5 to 53.5 m (3 samples) (Figure 2B). While the first niche is mainly characterised by a higher nitrate concentration, the second one differs from the third one mainly by (i) the concentration of particulate iron, phosphorus, magnesium, calcium, sulphur, and potassium, (ii) the concentration of dissolved sulphate, and (iii), the particulate organic carbon and nitrogen.

### Magnetotactic Bacteria Populations Are Diverse and Predominantly Composed of Magnetotactic Cocci

**FIGURE 3:**
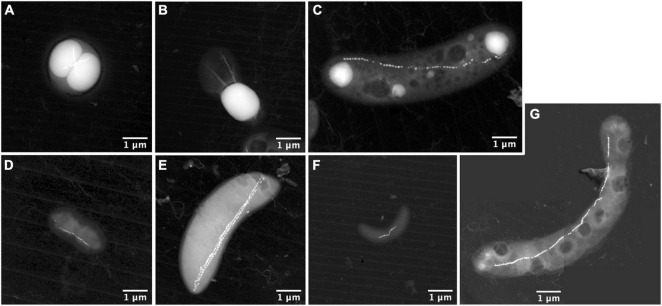
STEM-HAADF images of the different magnetotactic bacteria morphologies thriving in the Lake Pavin water column. It includes MTBc cells of the *Magnetococcaceae* family producing polyphosphate inclusions **(A)**; calcium-carbonate producing MTB **(B)**; rod-shaped MTB producing polyphosphate inclusions and affiliated to the *Magnetococcaceae* family **(C)**; small vibrio resembling the *Desulfovibrio magneticus* RS-1 bacteria with bullet-shaped magnetosomes **(D)**; rods resembling sulphate-reducing MTB producing bullet-shaped magnetosomes affiliated to the Deltaproteobacteria **(E)**; small vibrio resembling some species of the *Magnetospirillum* genus **(F)**; and long curved rod forming hexagonal magnetic crystals **(G)**.

**FIGURE 4:**
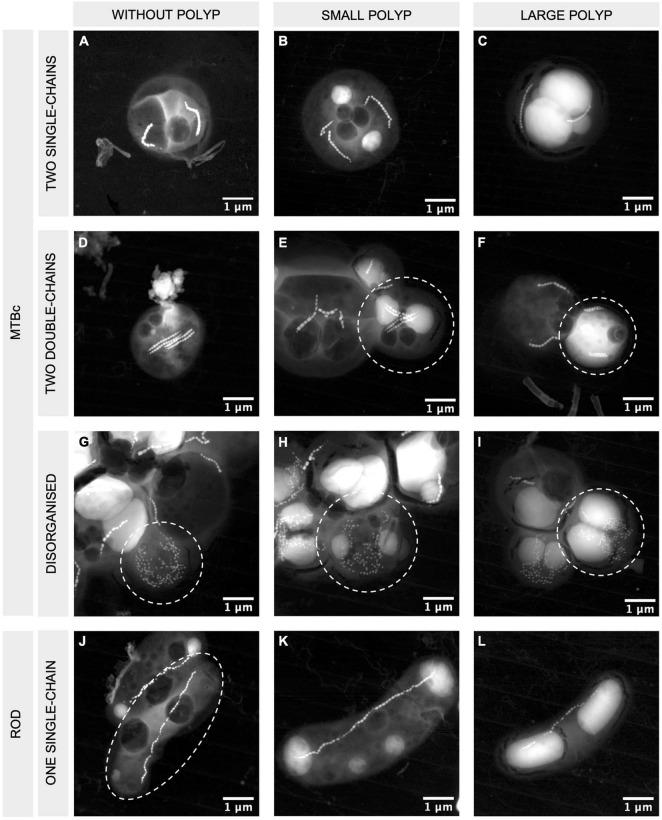
STEM-HAADF images of MTBc and rod-shaped MTB with different magnetosome organisations and different PolyP accumulation capabilities. MTBc with two single chains **(A–C)**, two double chains **(D–F)**, disorganised magnetosomes **(G–I)**, and rod-shaped MTB with one single chain of magnetosomes **(J–L)**. Columns from left to right are presenting MTB without PolyP, small PolyP, and large PolyP. The bacteria of interest are encircled in white. More information on the orientation of the crystals and their high-resolution images are given in Supplementary Figure 3.

**FIGURE 5:**
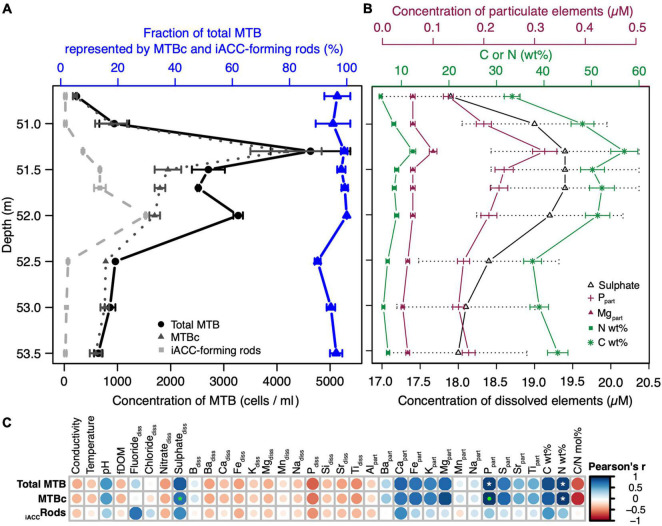
Relationships between the MTB population structure and abundance and the environmental features in the water column. **(A)** Cell concentration of the total MTB and those of the two main morphotypes, i.e., iACC-forming rods and MTBc, for the nine sampled depths. The fraction of total MTB represented by these two morphotypes are given for each depth. Error bars represent the standard deviation on biological duplicates (see Supplementary Table 1 for details). **(B)** Concentration profiles of the different geochemical variables significantly and positively correlated with either the total MTB or/and MTBc using Pearson’s and Spearman’s correlation tests. Particulate organic N and C are expressed in weight percent (wt%) of the total particulate organic matter. Error bars represent the maximal absolute error on measurement (±5% for dissolved sulphate and particulate organic N and C, ±7.5% for particulate P and Mg, respectively). **(C)** Statistical analysis of the correlation between the environmental variables and the cell concentrations of total MTB, MTBc, and iACC-forming rods. The plot shows the Pearson’s correlation matrix. The size and colour of the circles are related to the correlation coefficient value (or *r*), meaning that, the bigger and the darker the circle, the better the correlation. Blue and red circles refer to positive and negative correlations, respectively. The white and the green stars indicate significant linear correlations (Pearson’s test) or monotonic correlations (Spearman’s test), respectively. A green dot indicates that both tests were statistically significant. All statistics are given in Supplementary Table 2.

Seven different morphotypes were observed by electron microscopy across the nine depths (Figure 3). The MTBc, able to hyperaccumulate P under the form of PolyP, represented the most abundant morphotype (Figure 3A, XEDS supplied in Supplementary Figures 2A, 3), reaching between 52 and 94% of the total MTB population (Supplementary Table 1). MTBc cells were classified into three categories according to the organisation of their magnetosomes (Figure 4): (i) two single chains, (ii) two double chains, or (iii) disorganised magnetosomes. For all organisations, the magnetosome had a prismatic shape with a crystallographic elongation along the [111] direction of magnetite (Supplementary Figure 4). A large number of MTBc with empty vacuoles have been observed in samples at every depth in Lake Pavin, whatever their magnetosome organisation. Rod-shaped bacteria forming intracellular granules of amorphous calcium carbonate and prismatic magnetosomes represented the second most abundant MTB morphotype (Figure 3B, XEDS measurements supplied in Supplementary Figures 2B, 3). This morphotype, hereafter described as iACC-forming rods, represented between 2 and 47% of the total MTB population (Supplementary Table 1). Together with MTBc, they accounted for most of the total MTB population size representing between 88.5 and 98.8% of the MTB cells observed at each depth (Figure 5A).

To a minor extent, few other morphotypes could be observed in the deeper sampling zones where the populations of MTBc and iACC-forming rods are decreasing. One of these morphotypes also accumulating P as PolyP (Figure 3C, XEDS analyses supplied in Supplementary Figure 2C) is a rod with a chain of prismatic magnetosomes and has been previously affiliated to the *Magnetococcaceae* family (Monteil et al., 2021). Although this MTB morphotype is not very abundant in the water column of Lake Pavin, we observed a significant peak of abundance at 52.5-m depth. One type of small vibrio with ultrastructural features similar to those of *Desulfovibrio magneticus* RS-1 (Pósfai et al., 2006; Chariaou et al., 2015; Figure 3D) was also found along with another magnetotactic sulphate-reducing Deltaproteobacteria-*like* (Descamps et al., 2017) below 52.5 m (Figure 3E). Another small vibrio forming one chain of prismatic magnetosomes was detected at a low cell concentration as well (Figure 3F). The last morphotype of MTB was observed only once and appeared as a large curved rod, biomineralising a chain of prismatic magnetosomes (Figure 3G).

### The Abundance of Magnetotactic Cocci Is Correlated With the Concentration of Dissolved Sulphates, Particulate Organic N and C, Particulate Magnesium and Phosphorus

The structuration of the MTB communities was investigated along the water column in connection to geochemical niches. The MTB depth distribution was structured in two distinct peaks at 51.3- and 52-m depth (Figure 5A), where the total MTB cell concentration reached up to 4.6 × 10^3^ and 3.2 × 10^3^ cells per ml of water, respectively. While the cell concentration of MTBc was the highest at 51.3 m, that of the iACC-forming rods was the highest at 52-m depth.

Pairwise correlation analyses identified several geochemical variables strongly linked to the abundance of MTB populations (Figures 5B,C and Supplementary Table 2). The most striking results were obtained for the cell concentrations of MTBc and total MTB which were both significantly linearly correlated with the concentration of particulate phosphorus (Pearson’s correlation coefficients of 0.97 and 0.92, *p*-value = 2.0 × 10^–5^ and 3.7 × 10^–4^, respectively) and to the mass percentage of nitrogen (0.96 and 0.92, *p*-value = 5.9 × 10^–5^ and 4.4 × 10^–4^, respectively). A significant monotonic correlation was also observed between the concentration of dissolved sulphates and the cell concentration of MTBc (Spearman’s correlation coefficient of 0.95, *p*-value = 3.5 × 10^–4^). The variations on this geochemical parameter were low within the MTB zone (reaching approx. 1.5 μM, Figure 5B), but the same trend was obtained with the ICP-OES analyses for total dissolved sulphur (Supplementary Table 1). In addition, this weak but real sulphate peak was observed for previous sampling campaigns (Busigny et al., 2016; Miot et al., 2016; Berg et al., 2019).

Lowering the stringency on the significance level, the concentration of particulate magnesium appeared positively correlated with the cell concentration of MTBc (Pearson’s correlation coefficient of 0.89, *p*-value = 1.3 × 10^–3^), and the mass percentage of carbon appeared positively correlated with the cell concentrations of both total MTB and MTBc (Pearson’s correlation coefficients of 0.88 and 0.84, respectively, *p*-value < 5 × 10^–3^). Since MTBc represent the major MTB population in the water column, the profile of the MTBc cell concentration follows that of total MTB (Pearson’s correlation coefficient of 0.90, *p*-value = 0), and therefore, similar relationships were obtained for total MTB and MTBc cell concentrations (Figure 5C). No variable measured in this study was significantly correlated with the cell concentration of iACC-forming rods.

### The Proportion of Magnetotactic Cocci Sequestrating Polyphosphates Varies With Depth

**FIGURE 6:**
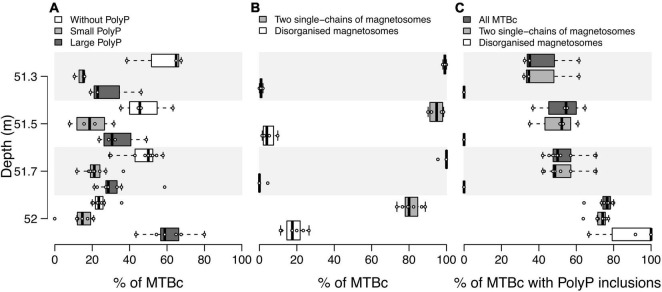
Distribution of MTBc in the water column according to their ability to form different types of magnetosome chains and PolyP inclusions. Boxplots showing the distribution over replicates of **(A)** the proportion of MTBc without, with small or with large PolyP inclusions for each sampled depth, **(B)** the proportion of MTBc with two single chains and disorganised magnetosomes independently of their capability to form PolyP, and **(C)** the proportion of cells with PolyP inclusions for MTBc with two single chains and disorganised magnetosomes. The numbers of counting replicates were as follows: *n* = 3, 4, 9, and 7 at 51.3, 51.5, 51.7, and 52.0 m depth, respectively (no replicate for the other depths, see section “Materials and Methods” for details). For each category of MTBc, (i) no significant difference was reported between their average proportions at 51. 3-, 51. 5-, and 51.7-m depth (pairwise Mann–Whitney–Wilcoxon test, *p*-value < 0.05), (ii) their average proportions significantly differ between 52-m depth and 51. 3-, 51. 5-, and 51.7-m depth, at the exception of the proportion of MTBc with small PolyP.

While iACC-forming rods are phenotypically homogeneous (i.e., they all produce inclusions filling most of their cytoplasm and exhibit the same magnetosome organisation), MTBc include different populations according to their PolyP content or their magnetosome organisation (Figure 4). Three categories of MTBc cells were defined according to the size of their PolyP inclusions: cells (i) without PolyP, (ii) with small PolyP, or (iii) with large PolyP inclusions, small and large referring here to inclusions filling less than and more than half of the cell area, respectively (Figure 4). MTBc cells harbour three different magnetosome organisations. Most of them had two single chains (from 82% at 52 m and up to 100% at 53 and 53.5 m), and few had disorganised magnetosomes (from 0% at 50.5, 51, 53, and 53.5 m and up to 17% at 52 m) (Figure 6B). Very rare MTBc with two double chains similar to those previously described (Zhang et al., 2017; Liu et al., 2020) were also observed (Figures 4D–F).

Population sizes (i.e., number of cells classified in a given population) determined on TEM grids were sometimes too limited to provide reliable counting replicates (see the section “Materials and Methods” for details). Considering all MTBc (independently of their magnetosome organisation) for the four water depths with counting replicates (from 51.3 to 52 m, Figure 6), our data suggest that the relative proportion of MTBc with large PolyP increases with depth and reaches a maximum at 52-m depth, while the opposite trend is observed for MTBc without PolyP (Figure 6A). Although MTBc with two single chains of magnetosomes represent the majority of the cells at these four depths, the proportion of MTBc with disorganised magnetosomes increases concomitantly with magnetotactic iACC-forming rods (at 52 m, Figure 6B). When classifying MTBc according to their magnetosome chains, it has to be noticed that the proportion of cells with large PolyP is increasing with depth, for each cell category (Figure 6C). STEM-HAADF observations also evidenced frequent empty vacuoles in each of the different categories of MTBc (Figure 4).

## Discussion

In this study, we evidenced a vertical stratification of MTB in the water column of Lake Pavin in October 2019, below the OATZ. It should be noticed that potential seasonal effects were out of the scope of this study. The MTBc, capable of sequestering large PolyP inclusions, dominated the MTB profile with a proportion comprised between 52 and 94% of all MTB at any depth in the MTB zone. The abundance of MTBc was correlated with the particulate organic carbon and nitrogen contents (Figure 5). The C/N molar ratio average value calculated in our samples is 6.63, which corresponds to the Redfield ratio for aquatic biomass (6.625) (Tyrrell, 2001). The ratio of MTBc *versus* other microorganisms in the water column of Lake Pavin only accounted to ∼1:1,000 (with abundance of bacteria and archaea estimated to 10^6^ cells ml^–1^ by Lehours et al., 2005). Rather than a causal impact of MTBc biomass on the organic N and C contents, these fractions of the particulate organic matter are more likely to correspond to a proxy for total biomass, previously evidenced to be represented at these depths by bacteria and archaea (Lehours et al., 2005). The optimal conditions for prokaryotic biomass development in this anoxic layer might also favour the MTBc growth.

Two main populations of MTBc with different magnetosome organisations were found along the water column of Lake Pavin with a maximum of abundance at different depths. MTBc with two single chains largely dominated the MTB abundance profile (>57%). The maximum abundance of this major morphotype was located at the same depth than the maximum of total MTB abundance (51.3 m). The minor MTBc morphotype with disorganised magnetosomes analogous to those described by, e.g., Koziaeva et al. (2019) and Liu et al. (2020), was most abundant at the same depth than the iACC-forming rods (52 m). Both MTBc morphotypes were located under anoxic conditions. The diversity of magnetosome organisations observed in MTBc suggests that there is more than one species of magnetotactic coccus in the water column of Lake Pavin. This hypothesis is based on a recent study demonstrating that MTBc populations with magnetosomes differing in number, crystal size, axial ratio, and chain configuration belong to different species (Liu et al., 2020). Moreover, this hypothesis is also reinforced by previous phylogenetic analyses made on Lake Pavin MTBc, which showed the presence of two different species based on the 16S rRNA gene sequence (Rivas-Lamelo et al., 2017).

The proportion of MTBc able to accumulate intracellular PolyP (from ∼35% at 51.3 m up to ∼75% at 52-m depth) was independent of the MTBc abundance. In addition, the PolyP sequestration capability was significantly higher at 52-m depth for both morphotypes of MTBc (i.e., two single chains and disorganised magnetosomes). This suggests that phosphorus hyperaccumulation capability depends on environmental conditions affecting both morphotypes.

Significant correlations were found between the concentration of dissolved sulphate and MTBc-related quantitative variables, providing clues about some metabolic traits of these bacteria (Figure 5). According to previous profiles of dissolved sulphate concentration monitored along the whole water column of Lake Pavin (Busigny et al., 2016; Miot et al., 2016), the local maximum of dissolved sulphate concentration observed within the MTB zone in this study corresponds to the global maximum within the water column. A positive correlation between the concentration of dissolved sulphate and the abundance of MTBc (as determined by light microscopy) could suggest that either sulphate is a preferred metabolic substrate for MTBc (e.g., for sulphate reduction) or a by-product of their metabolism (sulphoxidation) and/or of the metabolism of bacteria living in the same biotope. The observation of intracellular S granules suggests a sulphoxidation metabolism (Maki, 2013). At the cell scale, the TEM characterisation of inclusions of the major MTBc population (i.e., with two single chains of magnetosomes) suggests that cells accumulating large PolyP inclusions harbour none or small sulphur granules (Supplementary Figure 5). The reduced size of sulphur granules could be due to intracellular space limitations or to oxidation of the sulphur granules to gain energy for PolyP storage, the latter being previously evidenced by Kornberg (1995). It has been reported that P hyperaccumulators found in the environment are sulphur oxidisers (Brock and Schulz-Vogt, 2011; Möller et al., 2019). They have always been reported in the vicinity of an oxycline or within a gradient of sulphide concentration. For example, bacteria belonging to the *Sulfurimonas* genus were reported in an euxinic zone (anoxic and >0.1 μM of sulphide) of the water column of the Baltic Sea, where they need both nitrate and sulphide for their energy metabolism and to accumulate PolyP (Möller et al., 2019). In contrast, MTB affiliated to the *Magnetococcaceae* family from the suboxic layer (<0.1 μM oxygen and <0.1 μM sulphide) in the water column of the Black Sea store PolyP near the depth of oxygen disappearance and keep them under suboxic conditions until they hydrolyse them upon reaching the sulphide-rich zone of the water column (Schulz-Vogt et al., 2019). These two situations are distinct from the current paradigm based on *Beggiatoa* and *Thiomargarita* behaviours for which PolyP are accumulated under oxic conditions but are hydrolysed under both anoxic and sulphide-rich conditions (Schulz and Schulz, 2005; Brock and Schulz-Vogt, 2011). Similar to MTBc from the Black Sea and *Sulfurimonas* from the Baltic Sea, MTBc from Lake Pavin are also capable of keeping a high PolyP content in anoxic water. However, in contrast to the case observed in the Black Sea, the Lake Pavin phosphorus accumulators seem to accumulate PolyP not near the OATZ but rather deeper in the water column, close to the depth where the concentration of dissolved sulphide starts to increase (according to previously published profiles of total dissolved sulphide concentration in the water column, e.g., Michard et al., 1994; Bura-Nakić et al., 2009, 2013; Busigny et al., 2016; Rivas-Lamelo et al., 2017). Therefore, MTBc from Lake Pavin appear to have a metabolism closer to the *Sulfurimonas* from the Baltic Sea, for which sulphide is required to accumulate PolyP.

The nature and function of the empty vacuoles observed within MTBc at every depth in the water column of Lake Pavin are unclear. In the large sulphur-oxidising *Beggiatoa*, *Thiomargarita*, and *Thioploca*, empty vacuoles correspond to a nitrate-storing space for anoxic respiration and energy conservation (McHatton et al., 1996; Schulz, 1999; Zopfi et al., 2001). The closest relative to the Lake Pavin MTBc, the type strain *Magnetococcus marinus* MC-1 (Bazylinski et al., 2013), has been shown to harbour the set of genes required for denitrification (Philippot, 2002). Such metabolic capability has also been hypothesised for MTBc from the Black Sea (Schulz-Vogt et al., 2019). Moreover, some MTB affiliated to the Nitrospirae phylum also presenting empty vacuoles were suggested to couple sulphoxidation with denitrification (Li et al., 2020a). Thus, the MTBc of Lake Pavin could gain energy from their intracellular sulphur granules (i.e., sulphur oxidation) and potential intracellular nitrate (i.e., denitrification) for the accumulation of P as PolyP. The potential contribution of the MTBc metabolism to the nitrate depletion in the anoxic layer of the Lake Pavin water column (Supplementary Table 1; Miot et al., 2016) remains to be explored.

The sulphur/sulphide-oxidising bacteria (SOB) and sulphur/sulphate-reducing bacteria (SRB) are usually found around the redoxcline, in suboxic or euxinic waters (Vliet et al., 2020). In a recent study using 16S rRNA analyses in the water column of Lake Pavin, it has been shown that MTBc were at their highest abundance at the same depth where the SOB and SRB are both present, the former being dominant (Berg et al., 2019). The SRB become dominant deeper in the water column. Our observations evidenced by TEM the succession as a function of depth of (i) MTBc with two single chains of magnetosomes, followed by (ii) MTBc with disorganised magnetosomes, then more deeply (iii) the *Magnetococcaceae* rods accumulating PolyP, and (iv) potential sulphate-reducing Deltaproteobacteria, as suggested by their ultrastructure. These observations confirm the vertical succession of SOB and SRB in the water column. An important question is whether the succession of the three *Magnetococcaceae* morphotypes is due to competition or a difference of metabolism. The three of them accumulate sulphur granules intracellularly, suggesting they might all be SOB. Dissolved sulphate increases slightly around the MTBc abundance peak (Figure 5B). This increase could be due to oxidisation of intracellular sulphur storage by MTBc and species of the *Sulfuritalea* genus that represent the most abundant SOB in the water column (Berg et al., 2019). The *Sulfuritalea* are known to oxidise thiosulphate, S°, and hydrogen, but not sulphide, into sulphate as an end product. They can also use nitrate as an electron acceptor. Since some MTBc are known to use sulphide but *Sulfuritalea* do not, it might explain their coexistence at the same depths as they do not have to compete for electron donor but only for electron acceptor. Nitrate accumulated intracellularly could then be a good electron acceptor for the MTBc to thrive at these depths.

Previous studies of Lake Pavin showed an increase in the concentration of dissolved P below the OATZ reaching up to ∼300 μM deeper in the water column (Rivas-Lamelo et al., 2017). In the present study, this concentration starts to increase with depth immediately below the depth where the proportion of MTBc with PolyP was the highest. After a time of P deprivation when P becomes available again, some organisms such as microalgae have the ability to store more P than necessary in case of future shortage (Solovchenko et al., 2019); this ability is called luxury uptake. In the environmental context of Lake Pavin, we suggest that MTBc harbour a dynamic movement within their habitat. Upon reaching waters with a higher P concentration when moving downward, MTBc might store PolyP to a greater extent, preventing future P deprivation. MTBc might swim back upward towards more optimum conditions (i.e., at the depth where they are the most abundant) where they might hydrolyse their PolyP inclusions, as suggested by a lower proportion of MTBc with large PolyP. The imbalance between synthesis and hydrolysis of PolyP by MTBc remains to be evaluated along the water column. In Li and Dittrich (2019), cyanobacteria were shown to harbour a high content of PolyP during the lag phase due to an “overplus” uptake of P. By contrast, the amount of PolyP was lower during the exponential growth as a result of a competition between the P luxury uptake and its utilisation for growth. Similarly, MTBc could present a lower amount of PolyP at their peak of abundance (e.g., at 51.3 m), due to active growth, whereas they may contain higher amounts of PolyP deeper in the water column (e.g., at 52 m), where they might be in a growth dynamic closer to that of a lag phase. This hypothesis would support the luxury uptake hypothesis for these MTBc in combination with their dynamic movement along the water column.

Particulate Mg and P were found to be significantly correlated with the MTBc abundance (Figures 5B,C). PolyP are composed of negatively charged molecules and are commonly associated with counter ions with a valence of either +1 or +2 (Peverly et al., 1978; Kulaev et al., 2004). Our XEDS results showed that magnesium was a major component of PolyP in Lake Pavin (Supplementary Figures 2, 3). Therefore, at least at these depths, PolyP might be a major carrier of particulate Mg explaining the correlation of particulate Mg with the MTBc. This association has already been reported in MTBc from Lake Pavin (Rivas-Lamelo et al., 2017). The association of PolyP with Mg has also been shown for hyperaccumulators of P, e.g., *Sulfurimonas* in the Baltic Sea (Möller et al., 2019) and the giant sulphur-oxidising *Beggiatoa* (Brock et al., 2012). In addition to Mg as counter ion for PolyP in our samples, two other counter ions with a lower abundance were found to be associated with the PolyP, i.e., potassium and calcium. These two counter ions were shown to be significantly and positively correlated together.

In Lake Pavin, particulate P below the OATZ was previously evidenced to be hugely dominated by extracellular iron–phosphate particles, driving the turbidity profile (Cosmidis et al., 2014; Busigny et al., 2016; Rivas-Lamelo et al., 2017). The correlation between the MTBc abundance and the particulate P observed in the present study indicates that the MTB habitats overlap with the phosphogenesis zone. The systematic observation of cells encrusted within Fe–P minerals at these depths was formerly hypothesised as the result of several mechanisms, including PolyP hydrolysis or iron oxidation by microorganisms (Cosmidis et al., 2014; Miot et al., 2016; Berg et al., 2019). The observation of MTBc with low capability to accumulate intracellular PolyP in the phosphogenesis zone supports this hypothesis of PolyP release. The contribution of the dynamics of the P metabolism of MTBc to the phosphogenesis, occurring in the anoxic layer of the water column of Lake Pavin, remains to be determined.

**FIGURE 7:**
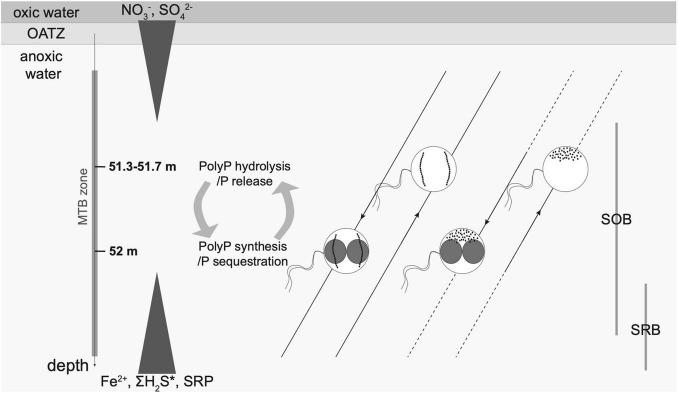
Schematic illustration of the main MTBc morphotypes inhabiting the anoxic layer of the water column of Lake Pavin. Six dominant morphotypes were defined according to TEM observations: with two single chains or disorganised magnetosomes and without, with small PolyP or with large PolyP (as presented in Figure 5). For each magnetosome organisation (two single chains and disorganised magnetosomes), the dominant MTBc morphotypes (without or with large PolyP, see relative proportions in Figure 6) are represented as a function of depth. Main geochemical gradients occurring along the water column are depicted by white triangles as follows. The concentration of dissolved nitrate begins at 0 μM for about the first 20 m of the water column, then increases to reach a maximum around 19 μM at the beginning of the MTB zone (50.7 m) and then decreases back down to the detection limit by the end of the MTB zone (Miot et al., 2016; Supplementary Table 1). Sulphate concentrations are nearly constant from the lake surface to the redox boundary and reach a maximum concentration (∼20 μM) in the middle of the MTB zone (51.5 m) before decreasing importantly with increasing depth to reach 0 μM (Miot et al., 2016; Figure 3B; Supplementary Table 1). Dissolved Fe, P (SRP), and H_2_S concentrations are undetectable from the surface of the water column down to the MTB zone and then progressively increase with depth up to ∼1,200, ∼300, and ∼22 μM towards the bottom of the water column, respectively (Rivas-Lamelo et al., 2017; Supplementary Table 1). Sulphur/sulphide-oxidising and sulphur/sulphate-reducing bacteria (SOB and SRB, respectively) are located as evidenced by Berg et al. (2019). Two single chains and disorganised magnetosomes are represented by intracellular black dots. PolyP inclusions are represented as unscaled red inclusions. MTBc with sulphur inclusions represented only a small fraction of the MTBc in the water column and are not displayed on the figure. The white lines represent the geomagnetic field lines, the white arrows indicating the direction. The dotted lines indicate the depth for which the MTBc cell concentrations were under the detection threshold.

Our results illustrate that magnetotaxis in MTBc of the Lake Pavin water column may not function in conjunction with aerotaxis, unlike for most known microaerophilic MTB that find efficiently their optimal oxygen concentration thanks to a magnetically assisted aerotaxis, the so-called magneto-aerotaxis (Frankel et al., 1997; Bennet et al., 2014; Lefèvre et al., 2014). MTBc would rather take advantage of their orientation with the Earth’s magnetic field to swim in one direction using the redox gradient to swim back and forth between their biotopes of interest, in particular to support their P and S metabolisms. In MTBc, magnetosome formation would thus represent a selective advantage to use magnetotaxis in conjunction with redoxtaxis (i.e., magnetically assisted redoxtaxis, or magneto-redoxtaxis) to efficiently migrate to and maintain the position at their preferred redox conditions (Figure 7).

It would be interesting in the future to better understand the P fluxes between the MTBc and the local environment and thus determine the conditions of active storage or hydrolysis of intracellular PolyP by MTBc. Their PolyP metabolism could also be explored by establishing the gene expression profiles in the water column using transcriptomics and evaluating the potential relations with P sequestration or hydrolysis. Lake Pavin represents a very promising natural laboratory to understand not only how PolyP are formed but also other types of biomineralisation such as iACC or magnetosomes. This study cannot identify a specific biogeochemical niche for the iACC-forming rods or the other minor morphotypes of MTB. The reasons could be that (i) a high biomass concentration is necessary for efficient statistical analyses along the entire MTB profile and (ii) parameters which structure their vertical distribution were not measured (e.g., pCO_2_). Future metagenomic studies will be helpful in order to reveal the different populations of MTB present in the water column of Lake Pavin and their functional diversity and will also give insight into the molecular pathways involved in the MTBc P and S metabolisms. We thus believe that our results are paving the way for future studies on other magnetotactic microorganisms that have this unique advantage to be magnetically concentrated and present a metabolic, taxonomic, and ultrastructural diversity of interest for a large community of scientists.

## Data Availability Statement

The original contributions presented in the study are included in the article/Supplementary Material, further inquiries can be directed to the corresponding author.

## Author Contributions

CB, CM, CL, NM, and ÉD designed the experiments. All authors contributed to water sampling and manuscript revision. CB and NM acquired and interpreted the STEM and XEDS data. CL and VB described the MTB morphotype diversity and abundance using light microscopy. DJ and ÉV acquired the physicochemical profiles and performed the chemical analyses of dissolved and particulate fractions. CM performed the statistical analyses. CB, CM, and ÉD wrote the manuscript.

## Conflict of Interest

The authors declare that the research was conducted in the absence of any commercial or financial relationships that could be construed as a potential conflict of interest.

## Publisher’s Note

All claims expressed in this article are solely those of the authors and do not necessarily represent those of their affiliated organizations, or those of the publisher, the editors and the reviewers. Any product that may be evaluated in this article, or claim that may be made by its manufacturer, is not guaranteed or endorsed by the publisher.

## References

[B1] Achbergerová L. Nahálka J. (2011). Polyphosphate - an ancient energy source and active metabolic regulator. *Microb. Cell Fact.* 10:63. 10.1186/1475-2859-10-63 21816086 PMC3163519

[B2] Amor M. Busigny V. Durand-Dubief M. Tharaud M. Ona-Nguema G. Gélabert A. (2015). Chemical signature of magnetotactic bacteria. *Proc. Natl. Acad. Sci. U.S.A.* 112 1699–1703. 10.1073/pnas.1414112112 25624469 PMC4330721

[B3] Amor M. Ceballos A. Wan J. Simon C. P. Aron A. T. Chang C. J. (2020). Magnetotactic bacteria accumulate a large pool of iron distinct from their magnetite crystals. *Biorxiv [Preprint]* 10.1101/2020.03.10.986679 PMC7642088 32887716

[B4] Bazylinski D. A. Dean A. J. Williams T. J. Long L. K. Middleton S. L. Dubbels B. L. (2004). Chemolithoautotrophy in the marine, magnetotactic bacterial strains MV-1 and MV-2. *Arch. Microbiol.* 182 373–387. 10.1007/s00203-004-0716-y 15338111

[B5] Bazylinski D. A. Williams T. J. Lefevre C. T. Berg R. J. Zhang C. L. Bowser S. S. (2013). *Magnetococcus marinus* gen. nov., sp. nov., a marine, magnetotactic bacterium that represents a novel lineage (Magnetococcaceae fam. nov., Magnetococcales ord. nov.) at the base of the Alphaproteobacteria. *Int. J. Syst. Evol. Microbiol.* 63 801–808. 10.1099/ijs.0.038927-0 22581902

[B6] Bennet M. McCarthy A. Fix D. Edwards M. R. Repp F. Vach P. (2014). Influence of magnetic fields on magneto-aerotaxis. *PLoS One* 9:e101150. 10.1371/journal.pone.0101150 24983865 PMC4077765

[B7] Benzerara K. Bolzoni R. Monteil C. Beyssac O. Forni O. Alonso B. (2020). The gammaproteobacterium *Achromatium* forms intracellular amorphous calcium carbonate and not (crystalline) calcite. *Geobiology* 19 199–213. 10.1111/gbi.12424 33347698

[B8] Berg J. S. Jézéquel D. Duverger A. Lamy D. Laberty-Robert C. Miot J. (2019). Microbial diversity involved in iron and cryptic sulfur cycling in the ferruginous, low-sulfate waters of Lake Pavin. *PLoS One* 14:e0212787. 10.1371/journal.pone.0212787 30794698 PMC6386445

[B9] Bonting C. F. Kortstee G. J. Zehnder A. J. (1991). Properties of polyphosphate: AMP phosphotransferase of Acinetobacter strain 210A. *J. Bacteriol.* 173 6484–6488. 10.1128/JB.173.20.6484-6488.1991 1655714 PMC208984

[B10] Bowlin M. Q. Gray M. J. (2021). Inorganic polyphosphate in host and microbe biology. *Trends Microbiol.* 29 1013–1023. 10.1016/j.tim.2021.02.002 33632603 PMC8380261

[B11] Brock J. Schulz-Vogt H. N. (2011). Sulfide induces phosphate release from polyphosphate in cultures of a marine *Beggiatoa* strain. *ISME J.* 5 497–506. 10.1038/ismej.2010.135 20827290 PMC3105714

[B12] Brock J. Rhiel E. Beutler M. Salman V. Schulz-Vogt H. N. (2012). Unusual polyphosphate inclusions observed in a marine *Beggiatoa* strain. *Antonie van Leeuwenhoek* 101 347–357. 10.1007/s10482-011-9640-8 21909788 PMC3261416

[B13] Bura-Nakić E. Viollier E. Ciglenečki I. (2013). Electrochemical and colorimetric measurements show the dominant role of FeS in a permanently anoxic lake. *Environ. Sci. Technol.* 47 741–749. 10.1021/es303603j 23240551

[B14] Bura-Nakić E. Viollier E. Jézéquel D. Thiam A. Ciglenečki I. (2009). Reduced sulfur and iron species in anoxic water column of meromictic crater Lake Pavin (Massif Central, France). *Chem. Geol.* 266 311–317. 10.1016/j.chemgeo.2009.06.020

[B15] Busigny V. Jézéquel D. Cosmidis J. Viollier E. Benzerara K. Planavsky N. J. (2016). “The Iron Wheel in Lac Pavin: interaction with phosphorus cycle,” in *Lake Pavin*, eds Sime-Ngando T. Boivin P. Chapron E. Jezequel D. Meybeck M. (Cham: Springer International Publishing), 205–220. 10.1007/978-3-319-39961-4_12

[B16] Busigny V. Mathon F. P. Jézéquel D. Bidaud C. C. Viollier E. Bardoux G. (2021). Mass collection of magnetotactic bacteria from the permanently stratified ferruginous Lake Pavin, France. *Environ. Microbiol.* 10.1111/1462-2920.15458 Online ahead of print 33687779

[B17] Cam N. Benzerara K. Georgelin T. Jaber M. Lambert J.-F. Poinsot M. (2016). Selective uptake of alkaline earth metals by cyanobacteria forming intracellular carbonates. *Environ. Sci. Technol.* 50 11654–11662. 10.1021/acs.est.6b02872 27712057

[B18] Chariaou M. Rahn-Lee L. Kind J. García-Rubio I. Komeili A. Gehring A. U. (2015). Anisotropy of bullet-shaped magnetite nanoparticles in the magnetotactic bacteria *Desulfovibrio magneticus* sp. Strain RS-1. *Biophys. J.* 108 1268–1274. 10.1016/j.bpj.2015.01.007 25762338 PMC4375454

[B19] Chen A. P. Berounsky V. M. Chan M. K. Blackford M. G. Cady C. Moskowitz B. M. (2014). Magnetic properties of uncultivated magnetotactic bacteria and their contribution to a stratified estuary iron cycle. *Nat. Commun.* 5:4797. 10.1038/ncomms5797 25175931

[B20] Chen H. Li J. Xing X. Du Z. Chen G. (2015). Unexpected Diversity of Magnetococci in Intertidal Sediments of Xiaoshi Island in the North Yellow Sea. *J. Nanomat.* 2015:902121. 10.1155/2015/902121

[B21] Cosmidis J. Benzerara K. Morin G. Busigny V. Lebeau O. Jézéquel D. (2014). Biomineralization of iron-phosphates in the water column of Lake Pavin (Massif Central, France). *Geochim. Cosmochim. Acta* 126 78–96. 10.1016/j.gca.2013.10.037

[B22] Couradeau E. Benzerara K. Gerard E. Moreira D. Bernard S. Brown G. E. (2012). An early-branching microbialite cyanobacterium forms intracellular carbonates. *Science* 336 459–462. 10.1126/science.1216171 22539718

[B23] Cox B. L. Popa R. Bazylinski D. A. Lanoil B. Douglas S. Belz A. (2002). Organization and elemental analysis of P-, S-, and Fe-rich inclusions in a population of freshwater magnetococci. *Geomicrobiol. J.* 19 387–406. 10.1080/01490450290098504

[B24] Crosby C. H. Bailey J. V. (2012). The role of microbes in the formation of modern and ancient phosphatic mineral deposits. *Front. Microbiol.* 3:241. 10.3389/fmicb.2012.00241 22783245 PMC3389779

[B25] Descamps E. C. T. Monteil C. L. Menguy N. Ginet N. Pignol D. Bazylinski D. A. (2017). *Desulfamplus magnetovallimortis* gen. nov., sp. nov., a magnetotactic bacterium from a brackish desert spring able to biomineralize greigite and magnetite, that represents a novel lineage in the *Desulfobacteraceae*. *Syst. Appl. Microbiol.* 40 280–289. 10.1016/j.syapm.2017.05.001 28622795

[B26] Eder S. H. K. Gigler A. M. Hanzlik M. Winklhofer M. (2014). Sub-micrometer-scale mapping of magnetite crystals and sulfur globules in magnetotactic bacteria using confocal raman micro-spectrometry. *PLoS One* 9:e107356. 10.1371/journal.pone.0107356 25233081 PMC4169400

[B27] Frankel R. B. Bazylinski D. A. Johnson M. S. Taylor B. L. (1997). Magneto-aerotaxis in marine coccoid bacteria. *Biophys. J.* 73 994–1000. 10.1016/S0006-3495(97)78132-3 9251816 PMC1180996

[B28] Harrell F. E. Jr. (2020). *Hmisc: Harrell Miscellaneous. R Package Version 4.4-0.*

[B29] Head I. M. Gray N. D. Clarke K. J. Pickup R. W. Jones J. G. (1996). The phylogenetic position and ultrastructure of the uncultured bacterium *Achromatium oxaliferum*. *Microbiology* 142 2341–2354. 10.1099/00221287-142-9-2341 8828202

[B30] Isambert A. Menguy N. Larquet E. Guyot F. Valet J.-P. (2007). Transmission electron microscopy study of magnetites in a freshwater population of magnetotactic bacteria. *Am. Mineral.* 92 621–630. 10.2138/am.2007.2278

[B31] Ji B. Zhang S.-D. Zhang W.-J. Rouy Z. Alberto F. Santini C.-L. (2017). The chimeric nature of the genomes of marine magnetotactic coccoid-ovoid bacteria defines a novel group of *Proteobacteria*: genome of marine magnetotactic strain MO-1. *Environ. Microbiol.* 19 1103–1119. 10.1111/1462-2920.13637 27902881

[B32] Kampinga H. H. (2014). Chaperoned by prebiotic inorganic polyphosphate molecules: an ancient transcription-independent mechanism to restore protein homeostasis. *Mol. Cell* 53 685–687. 10.1016/j.molcel.2014.02.023 24606917

[B33] Kawakoshi A. Nakazawa H. Fukada J. Sasagawa M. Katano Y. Nakamura S. (2012). Deciphering the genome of polyphosphate accumulating actinobacterium microlunatus phosphovorus. *DNA Res.* 19 383–394. 10.1093/dnares/dss020 22923697 PMC3473371

[B34] Keim C. N. Duarte de Melo R. Almeida F. P. Lins de Barros H. G. P. Farina M. (2018). Effect of applied magnetic fields on motility and magnetotaxis in the uncultured magnetotactic multicellular prokaryote ‘*Candidatus* magnetoglobus multicellularis’: magnetotaxis in multicellular prokaryotes. *Environ. Microbiol. Rep.* 10 465–474. 10.1111/1758-2229.12640 29573371

[B35] Klumpp S. Lefèvre C. T. Bennet M. Faivre D. (2019). Swimming with magnets: from biological organisms to synthetic devices. *Phys. Rep.* 789 1–54. 10.1016/j.physrep.2018.10.007

[B36] Kobayashi A. Kirschvink J. L. Nash C. Z. Kopp R. E. Sauer D. A. Bertani L. E. (2006). Experimental observation of magnetosome chain collapse in magnetotactic bacteria: sedimentological, paleomagnetic, and evolutionary implications. *Earth Planet. Sci. Lett.* 245 538–550. 10.1016/j.epsl.2006.03.041

[B37] Kornberg A. (1995). Inorganic polyphosphate: toward making a forgotten polymer unforgettable. *J. Bacteriol.* 177 491–496. 10.1128/JB.177.3.491-496.1995 7836277 PMC176618

[B38] Kornberg A. Rao N. N. Ault-Riché D. (1999). Inorganic polyphosphate: a molecule of many functions. *Annu. Rev. Biochem.* 68 89–125. 10.1146/annurev.biochem.68.1.89 10872445

[B39] Koziaeva V. Dziuba M. Leão P. Uzun M. Krutkina M. Grouzdev D. (2019). Genome-based metabolic reconstruction of a novel uncultivated freshwater magnetotactic coccus “Ca. Magnetaquicoccus inordinatus” UR-1, and Proposal of a Candidate Family “Ca. Magnetaquicoccaceae.”. *Front. Microbiol.* 10:2290. 10.3389/fmicb.2019.02290 31632385 PMC6783814

[B40] Kristiansen R. Nguyen H. T. T. Saunders A. M. Nielsen J. L. Wimmer R. Le V. Q. (2013). A metabolic model for members of the genus *Tetrasphaera* involved in enhanced biological phosphorus removal. *ISME J.* 7 543–554. 10.1038/ismej.2012.136 23178666 PMC3578573

[B41] Kulaev I. S. (1975). Biochemistry of inorganic polyphosphates. *Rev. Physiol. Biochem. Pharmacol.* 86 131–158. 10.1007/BFb0034661 175427

[B42] Kulaev I. S. Vagabov V. M. Kulakovskaya T. V. (2004). *The Biochemistry of Inorganic Polyphosphates*, 2nd Edn. Hoboken, NJ: Wiley.

[B43] Lê S. Josse J. Husson F. (2008). FactoMineR: an r package for multivariate analysis. *J. Stat. Soft.* 25 1–18. 10.18637/jss.v025.i01

[B44] Lechaire J.-P. Shillito B. Frébourg G. Gaill F. (2002). Elemental characterization of microorganism granules by EFTEM in the tube wall of a deep-sea vent invertebrate. *Biol. Cell* 94 243–249. 10.1016/S0248-4900(02)01199-1 12489693

[B45] Lefevre C. T. Bazylinski D. A. (2013). Ecology, diversity, and evolution of magnetotactic bacteria. *Microbiol. Mol. Biol. Rev.* 77 497–526. 10.1128/MMBR.00021-13 24006473 PMC3811606

[B46] Lefèvre C. T. Bennet M. Landau L. Vach P. Pignol D. Bazylinski D. A. (2014). Diversity of magneto-aerotactic behaviors and oxygen sensing mechanisms in cultured magnetotactic bacteria. *Biophys. J.* 107 527–538. 10.1016/j.bpj.2014.05.043 25028894 PMC4104051

[B47] Lefèvre C. T. Viloria N. Schmidt M. L. Pósfai M. Frankel R. B. Bazylinski D. A. (2012). Novel magnetite-producing magnetotactic bacteria belonging to the Gammaproteobacteria. *ISME J.* 6 440–450. 10.1038/ismej.2011.97 21776027 PMC3260515

[B48] Lehours A.-C. Bardot C. Thenot A. Debroas D. Fonty G. (2005). Anaerobic microbial communities in Lake Pavin, a unique meromictic Lake in France. *Appl. Environ. Microbiol.* 71 7389–7400. 10.1128/AEM.71.11.7389-7400.2005 16269781 PMC1287608

[B49] Li J. Dittrich M. (2019). Dynamic polyphosphate metabolism in cyanobacteria responding to phosphorus availability: polyphosphate in cyanobacteria. *Environ. Microbiol.* 21 572–583. 10.1111/1462-2920.14488 30474918

[B50] Li J. Ge K. Pan Y. Williams W. Liu Q. Qin H. (2013). A strong angular dependence of magnetic properties of magnetosome chains: implications for rock magnetism and paleomagnetism: magnetism of magnetosome chains. *Geochem. Geophys. Geosystems* 14 3887–3907. 10.1002/ggge.20228

[B51] Li J. Liu P. Tamaxia A. Zhang H. Liu Y. Wang J. (2021). Diverse intracellular inclusion types within magnetotactic bacteria: implications for biogeochemical cycling in aquatic environments. *J. Geophys. Res. Biogeosci*. 126:e2021JG006310. 10.1029/2021JG006310

[B52] Li J. Liu P. Wang J. Roberts A. P. Pan Y. (2020a). Magnetotaxis as an adaptation to enable bacterial shuttling of microbial sulfur and sulfur cycling across aquatic oxic-anoxic interfaces. *J. Geophys. Res. Biogeosci.* 125:e2020JG006012. 10.1029/2020JG006012

[B53] Li J. Menguy N. Leroy E. Roberts A. P. Liu P. Pan Y. (2020b). Biomineralization and magnetism of uncultured magnetotactic coccus strain THC-1 with non-chained magnetosomal magnetite nanoparticles. *J. Geophys. Res. Solid Earth* 125:e2020JB020853. 10.1029/2020JB020853

[B54] Lin W. Bazylinski D. A. Xiao T. Wu L.-F. Pan Y. (2014). Life with compass: diversity and biogeography of magnetotactic bacteria: magnetotactic bacterial diversity and biogeography. *Environ. Microbiol.* 16 2646–2658. 10.1111/1462-2920.12313 24148107

[B55] Lin W. Zhang W. Zhao X. Roberts A. P. Paterson G. A. Bazylinski D. A. (2018). Genomic expansion of magnetotactic bacteria reveals an early common origin of magnetotaxis with lineage-specific evolution. *ISME J.* 12 1508–1519. 10.1038/s41396-018-0098-9 29581530 PMC5955933

[B56] Lins U. Farina M. (1999). Phosphorus-rich granules in uncultured magnetotactic bacteria. *FEMS Microbiol. Lett.* 172 23–28. 10.1111/j.1574-6968.1999.tb13444.x

[B57] Liu P. Liu Y. Ren X. Zhang Z. Zhao X. Roberts A. P. (2021). A novel magnetotactic Alphaproteobacterium producing intracellular magnetite and calcium-bearing minerals. *Appl. Environ. Microbiol*. 87:e0155621. 10.1128/AEM.01556-21 34756060 PMC8579999

[B58] Liu P. Liu Y. Zhao X. Roberts A. P. Zhang H. Zheng Y. (2020). Diverse phylogeny and morphology of magnetite biomineralized by magnetotactic cocci. *Environ. Microbiol.* 23 1115–1129. 10.1111/1462-2920.15254 32985765

[B59] Maki J. S. (2013). Bacterial intracellular sulfur globules: structure and function. *J. Mol. Microbiol. Biotechnol.* 23 270–280. 10.1159/000351335 23920490

[B60] Mänd K. Kirsimäe K. Lepland A. Crosby C. H. Bailey J. V. Konhauser K. O. (2018). Authigenesis of biomorphic apatite particles from Benguela upwelling zone sediments off Namibia: the role of organic matter in sedimentary apatite nucleation and growth. *Geobiology* 16 640–658. 10.1111/gbi.12309 30062734

[B61] McHatton S. C. Barry J. P. Jannasch H. W. Nelson D. C. (1996). High nitrate concentrations in vacuolate, autotrophic marine *Beggiatoa* spp. *Appl. Environ. Microbiol.* 62 954–958. 10.1128/AEM.62.3.954-958.1996 16535282 PMC1388807

[B62] Michard G. Viollier E. Jézéquel D. Sarazin G. (1994). Geochemical study of a crater lake: Pavin Lake, France — Identification, location and quantification of the chemical reactions in the lake. *Chem. Geol.* 115 103–115. 10.1016/0009-2541(94)90147-3

[B63] Miot J. Jézéquel D. Benzerara K. Cordier L. Rivas-Lamelo S. Skouri-Panet F. (2016). Mineralogical diversity in Lake Pavin: connections with water column chemistry and biomineralization processes. *Minerals* 6:24. 10.3390/min6020024

[B64] Möller L. Laas P. Rogge A. Goetz F. Bahlo R. Leipe T. (2019). *Sulfurimonas* subgroup GD17 cells accumulate polyphosphate under fluctuating redox conditions in the Baltic Sea: possible implications for their ecology. *ISME J.* 13 482–493. 10.1038/s41396-018-0267-x 30291329 PMC6331637

[B65] Monteil C. L. Benzerara K. Menguy N. Bidaud C. C. Michot-Achdjian E. Bolzoni R. (2021). Intracellular amorphous Ca-carbonate and magnetite biomineralization by a magnetotactic bacterium affiliated to the Alphaproteobacteria. *ISME J.* 15 1–18. 10.1038/s41396-020-00747-3 32839547 PMC7853122

[B66] Monteil C. L. Perrière G. Menguy N. Ginet N. Alonso B. Waisbord N. (2018). Genomic study of a novel magnetotactic *Alphaproteobacteria* uncovers the multiple ancestry of magnetotaxis. *Environ. Microbiol.* 20 4415–4430. 10.1111/1462-2920.14364 30043533

[B67] Mußmann M. Hu F. Z. Richter M. de Beer D. Preisler A. Jørgensen B. B. (2007). Insights into the genome of large sulfur bacteria revealed by analysis of single filaments. *PLoS Biol.* 5:e230. 10.1371/journal.pbio.0050230 17760503 PMC1951784

[B68] Oestreicher Z. Lower S. Lower B. (2011). Magnetotactic bacteria containing phosphorus-rich inclusion bodies. *Microsc. Microanal.* 17 140–141. 10.1017/S1431927611001577

[B69] Oyserman B. O. Moya F. Lawson C. E. Garcia A. L. Vogt M. Heffernen M. (2016). Ancestral genome reconstruction identifies the evolutionary basis for trait acquisition in polyphosphate accumulating bacteria. *ISME J.* 10 2931–2945. 10.1038/ismej.2016.67 27128993 PMC5148189

[B70] Parks D. H. Chuvochina M. Waite D. W. Rinke C. Skarshewski A. Chaumeil P.-A. (2018). A standardized bacterial taxonomy based on genome phylogeny substantially revises the tree of life. *Nat. Biotechnol.* 36 996–1004. 10.1038/nbt.4229 30148503

[B71] Peverly J. H. Adamec J. Parthasarathy M. V. (1978). Association of potassium and some other monovalent cations with occurrence of polyphosphate bodies in *Chlorella pyrenoidosa*. *Plant Physiol.* 62 120–126. 10.1104/pp.62.1.120 16660449 PMC1092069

[B72] Philippot L. (2002). Denitrifying genes in bacterial and Archaeal genomes. *Biochimica Biophysica Acta* 1577 355–376. 10.1016/S0167-4781(02)00420-7 12359326

[B73] Popp F. Armitage J. P. Schüler D. (2014). Polarity of bacterial magnetotaxis is controlled by aerotaxis through a common sensory pathway. *Nat. Commun.* 5:5398. 10.1038/ncomms6398 25394370

[B74] Pósfai M. Moskowitz B. M. Arató B. Schüler D. Flies C. Bazylinski D. A. (2006). Properties of intracellular magnetite crystals produced by *Desulfovibrio magneticus* strain RS-1. *Earth Planet. Sci. Lett.* 249 444–455. 10.1016/j.epsl.2006.06.036

[B75] R Core Team (2020). *R: A Language and Environment for Statistical Computing.* Vienna: R Core Team.

[B76] Rivas-Lamelo S. Benzerara K. Lefèvre C. T. Monteil C. L. Jézéquel D. Menguy N. (2017). Magnetotactic bacteria as a new model for P sequestration in the ferruginous Lake Pavin. *Geochem. Perspect. Lett.* 5 35–41. 10.7185/geochemlet.1743 15074972

[B77] Salman V. Yang T. Berben T. Klein F. Angert E. Teske A. (2015). Calcite-accumulating large sulfur bacteria of the genus *Achromatium* in Sippewissett Salt Marsh. *ISME J.* 9 2503–2514. 10.1038/ismej.2015.62 25909974 PMC4611513

[B78] Schüler D. (1999). Formation of magnetosomes in magnetotactic bacteria. *J. Molec. Microbiol. Biotechnol.* 1 79–86. 10941788

[B79] Schüler D. (2002). The biomineralization of magnetosomes in *Magnetospirillum gryphiswaldense*. *Int. Microbiol.* 5 209–214. 10.1007/s10123-002-0086-8 12497187

[B80] Schulz H. N. (1999). Dense populations of a giant sulfur bacterium in namibian shelf sediments. *Science* 284 493–495. 10.1126/science.284.5413.493 10205058

[B81] Schulz H. N. Schulz H. D. (2005). Large sulfur bacteria and the formation of phosphorite. *Science* 307 416–418. 10.1126/science.1103096 15662012

[B82] Schulz-Vogt H. N. Pollehne F. Jürgens K. Arz H. W. Beier S. Bahlo R. (2019). Effect of large magnetotactic bacteria with polyphosphate inclusions on the phosphate profile of the suboxic zone in the Black Sea. *ISME J.* 13 1198–1208. 10.1038/s41396-018-0315-6 30643197 PMC6474215

[B83] Seufferheld M. J. Alvarez H. M. Farias M. E. (2008). Role of polyphosphates in microbial adaptation to extreme environments. *App. Environ. Microbiol.* 74 5867–5874. 10.1128/AEM.00501-08 18708516 PMC2565944

[B84] Sime-Ngando T. Boivin P. Chapron E. Jezequel D. Meybeck M. (eds) (2016). *Lake Pavin.* Cham: Springer International Publishing, 10.1007/978-3-319-39961-4

[B85] Solovchenko A. Khozin-Goldberg I. Selyakh I. Semenova L. Ismagulova T. Lukyanov A. (2019). Phosphorus starvation and luxury uptake in green microalgae revisited. *Algal Res.* 43:101651. 10.1016/j.algal.2019.101651

[B86] Tyrrell T. (2001). “Redfield ratio,” in *Encyclopedia of Ocean Sciences* (Cambridge, MA: Academic Press), 2377–2387. 10.1006/rwos.2001.0271

[B87] Uebe R. Schüler D. (2016). Magnetosome biogenesis in magnetotactic bacteria. *Nat. Rev. Microbiol.* 14 621–637. 10.1038/nrmicro.2016.99 27620945

[B88] Varela C. Mauriaca C. Paradela A. Albar J. P. Jerez C. A. Chávez F. P. (2010). New structural and functional defects in polyphosphate deficient bacteria: a cellular and proteomic study. *BMC Microbiol.* 10:7. 10.1186/1471-2180-10-7 20067623 PMC2817675

[B89] Vigneau E. Mingkun C. Qannari E. M. (2015). ClustVarLV: an R package for the clustering of variables around latent variables. *R J.* 7 134–148.

[B90] Vliet D. M. Meijenfeldt F. A. B. Dutilh B. E. Villanueva L. Sinninghe Damsté J. S. Stams A. J. M. (2020). The bacterial sulfur cycle in expanding dysoxic and euxinic marine waters. *Environ. Microbiol.* 23 2834–2857. 10.1111/1462-2920.15265 33000514 PMC8359478

[B91] Wang L. Yan J. Wise M. J. Liu Q. Asenso J. Huang Y. (2018). Distribution patterns of polyphosphate metabolism pathway and its relationships with bacterial durability and virulence. *Front. Microbiol.* 9:782. 10.3389/fmicb.2018.00782 29755430 PMC5932413

[B92] Wei T. Simko V. (2017). *R package “corrplot”: Visualization of a Correlation Matrix (Version 0.84).*

[B93] Zhang H. Menguy N. Wang F. Benzerara K. Leroy E. Liu P. (2017). Magnetotactic coccus strain SHHC-1 affiliated to alphaproteobacteria forms octahedral magnetite magnetosomes. *Front. Microbiol.* 8:969. 10.3389/fmicb.2017.00969 28611762 PMC5447723

[B94] Zopfi J. Kjær T. Nielsen L. P. Jørgensen B. B. (2001). Ecology of *Thioploca* spp.: nitrate and sulfur storage in relation to chemical microgradients and influence of *Thioploca* spp. on the sedimentary nitrogen cycle. *Appl. Environ. Microbiol.* 67 5530–5537. 10.1128/AEM.67.12.5530-5537.2001 11722903 PMC93340

