# Supplementary material for: Biogeochemical Niche of Magnetotactic Cocci Capable of Sequestering Large Polyphosphate Inclusions in the Anoxic Layer of the Lake Pavin Water Column
Source: Front Microbiol. 2022 Jan 10;12:789134. doi: 10.3389/fmicb.2021.789134 (PMC8786505; doi:10.3389/fmicb.2021.789134)
Supplement: Supplementary file 1 [file Data_Sheet_1.docx]

**
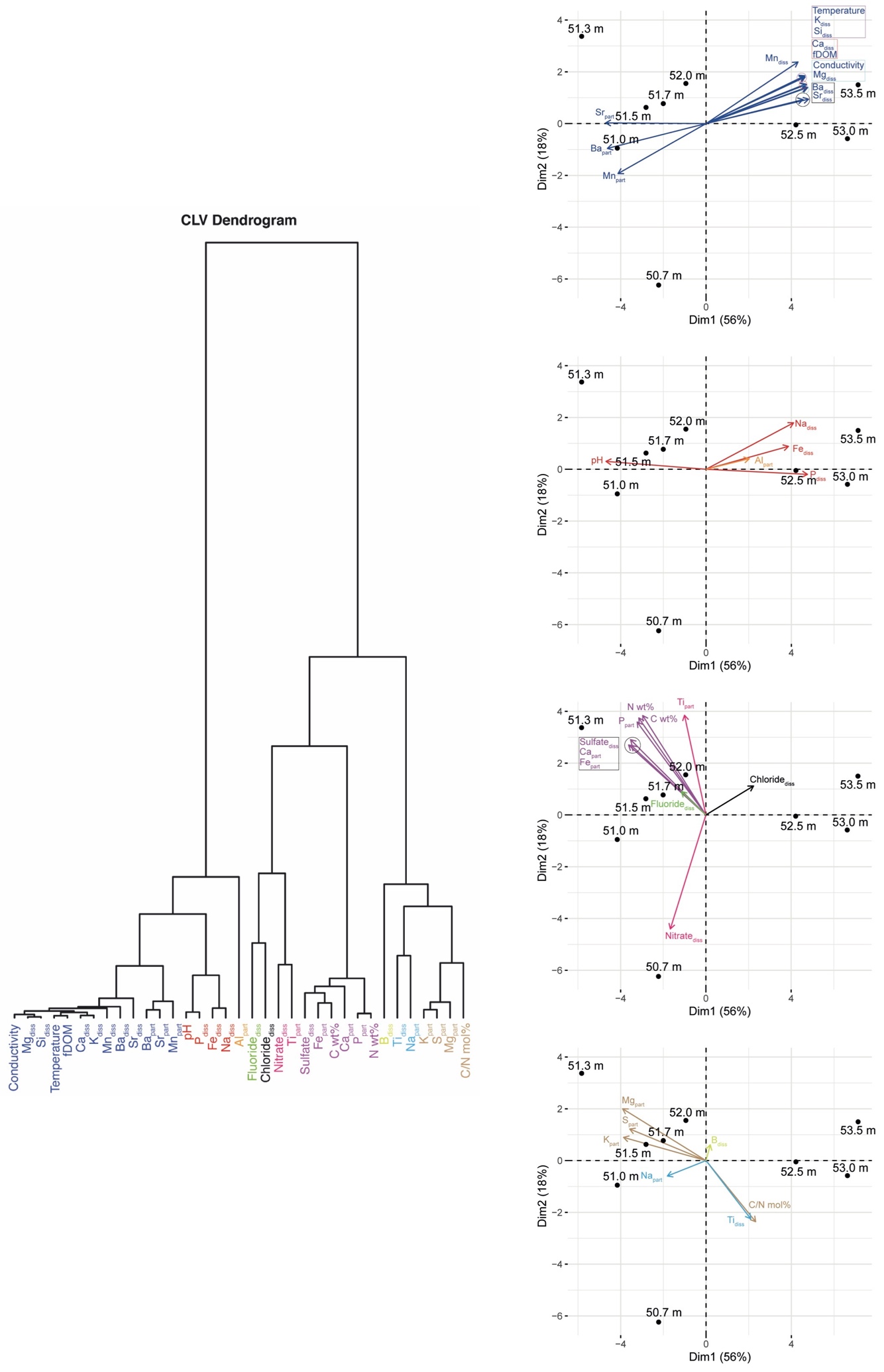
**

**Supplementary Figure S1.The ten directional groups of environmental parameters defined by hierarchical Clustering around Latent Variables (left) and represented in the PCA biplot (right).** The CLV dendrogram represents the hierarchical relationships between the 35 variables (i.e. the physicochemical and geochemical parameters, represented as tree leaves) describing the 9 water samples. The order by which the leaves are connected in the dendrogram is directly related to the strength of the linear correlation between the variables: the smaller the vertical distance between two leaves and the node connecting them, the stronger the correlation between the two corresponding variables. Optimal number of groups of correlated variables was inferred.
