# Supplementary material for: Biogeochemical Niche of Magnetotactic Cocci Capable of Sequestering Large Polyphosphate Inclusions in the Anoxic Layer of the Lake Pavin Water Column
Source: Front Microbiol. 2022 Jan 10;12:789134. doi: 10.3389/fmicb.2021.789134 (PMC8786505; doi:10.3389/fmicb.2021.789134)
Supplement: Supplementary file 2 [file Data_Sheet_2.docx]

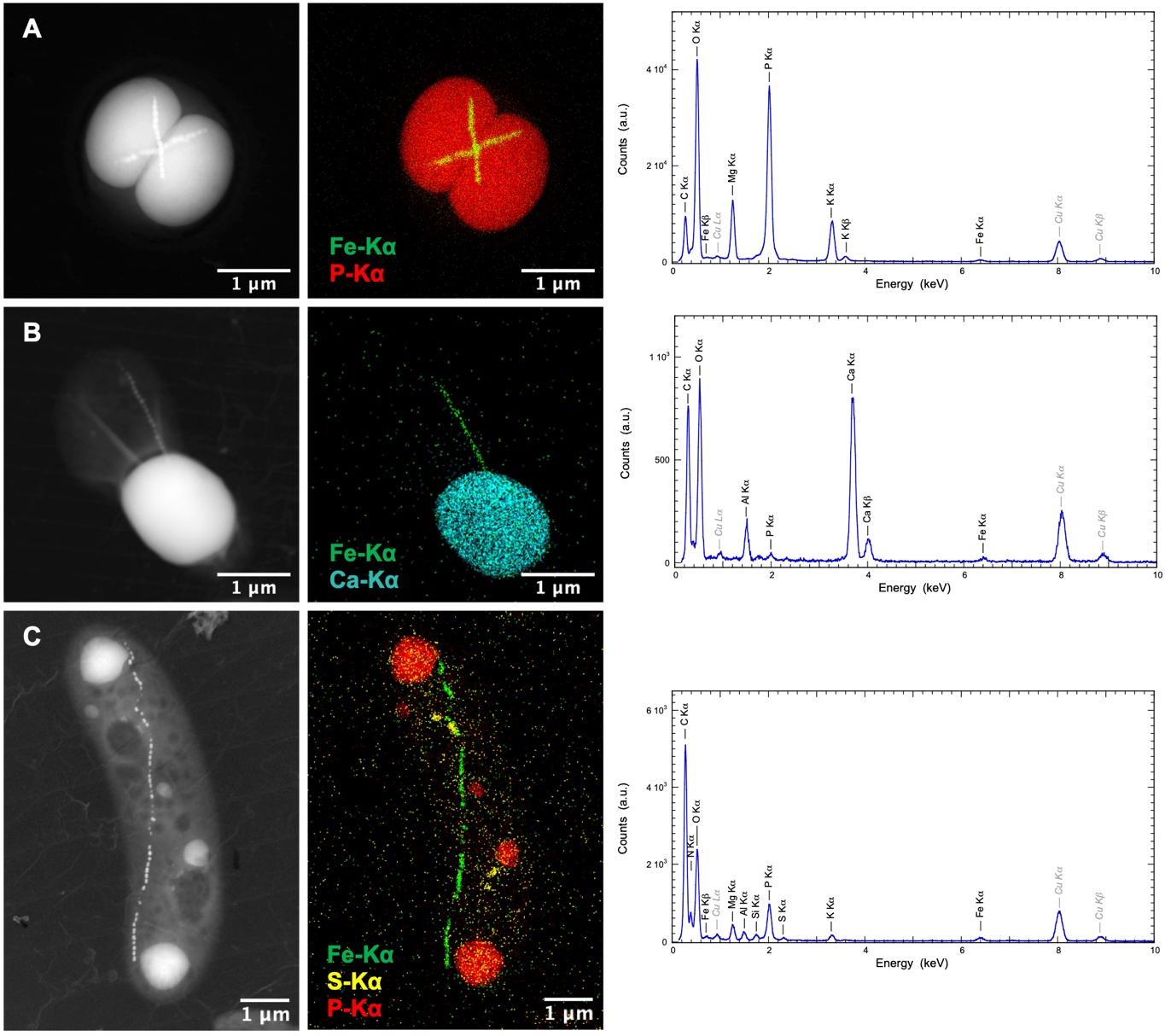


**Supplementary Figure S2.** **Elemental mapping of the intracellular inclusions of MTBc, iACC-forming rods and rod-shaped MTB as illustrated in Figure 3.** Each morphotype is characterised by a STEM-HAADF image, a composite elemental STEM-XEDS image and an X-ray fluorescence spectrum. (A) MTBc with large inclusions of PolyP (Fig. 3A). (B) iACC-forming rods (calcium carbonate inclusions, Fig 3B). (C) Rod-shaped MTB with PolyP inclusions (Fig. 3C).
