# Supplementary material for: Biogeochemical Niche of Magnetotactic Cocci Capable of Sequestering Large Polyphosphate Inclusions in the Anoxic Layer of the Lake Pavin Water Column
Source: Front Microbiol. 2022 Jan 10;12:789134. doi: 10.3389/fmicb.2021.789134 (PMC8786505; doi:10.3389/fmicb.2021.789134)
Supplement: Supplementary file 3 [file Data_Sheet_3.docx]

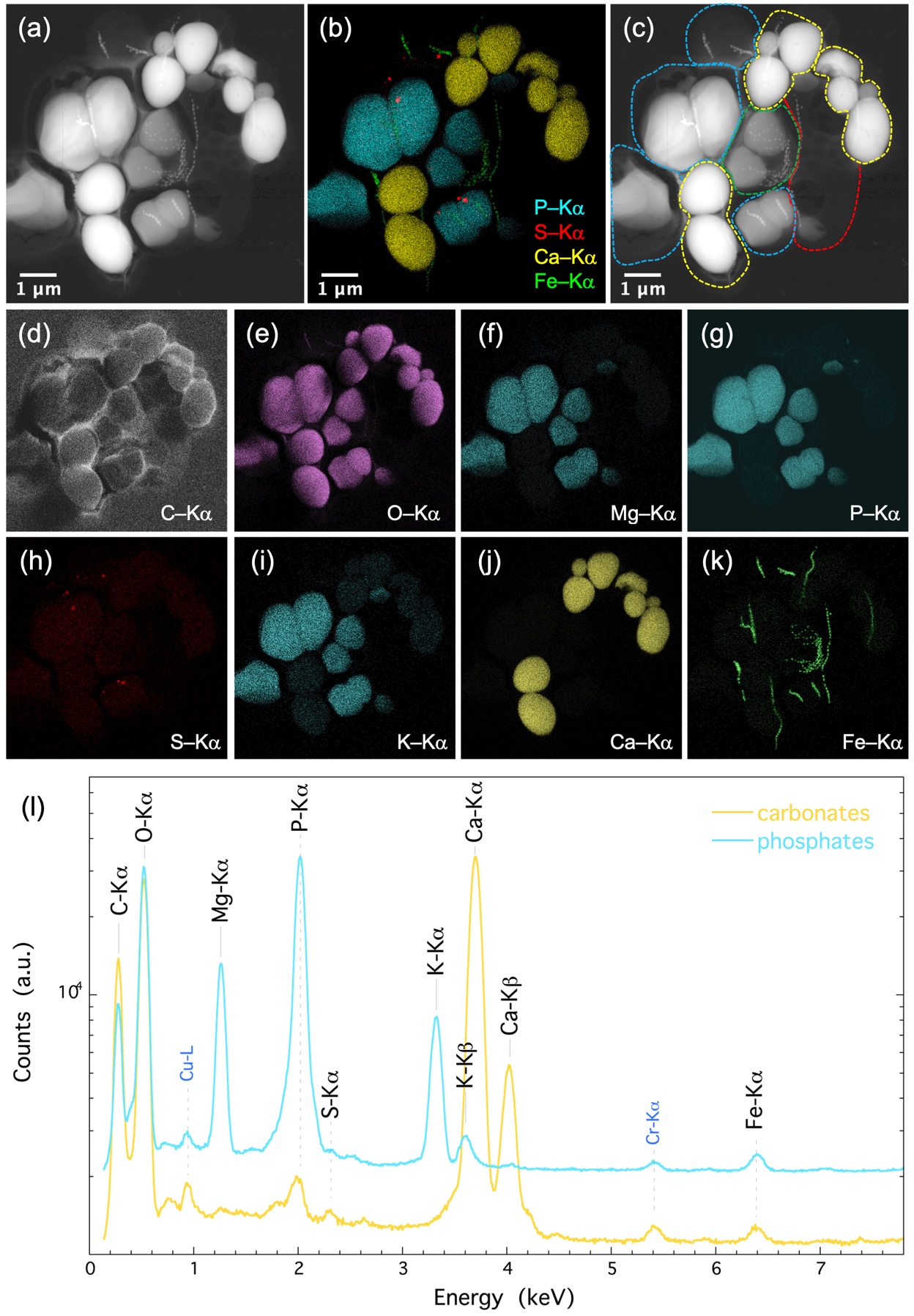


**Supplementary Figure S3.** **Elemental mapping of MTB intracellular inclusions (PolyP, S granules, iACC, magnetosomes).** (a) STEM-HAADF and (b) composite elemental image showing the coexistence of PolyP sequestering MTBc with two single-chains of magnetosomes (blue circle in (c)) or disorganised magnetosomes (green circle in (c)) and iACC-forming rods (yellow circle in (c)) MTB. Note also the coexistence of a MTB rod sequestering two small polar PolyP inclusions (red circle in (c)). (d-k) elemental STEM-XEDS mapping of major elements (X-ray lines are indicated). It has to be noticed that the images are not background corrected. For some images (Mg, P, S, K and Fe) apparent intensity may be due to Bremsstrahlung and is therefore not related to the actual presence of these elements. The Carbon C-Kα map is affected by absorption process and shadowing effect because of the X-ray detector orientation. (l) X-ray fluorescence spectra related to carbonates and phosphates, respectively (the second spectrum has been shifted for clarity). Intensity is plotted in logarithmic scale to emphasize the minor elements signal. Cu-L and Cr-Kα peaks are spurious signals due to the sample holder.
