# Supplementary material for: Biogeochemical Niche of Magnetotactic Cocci Capable of Sequestering Large Polyphosphate Inclusions in the Anoxic Layer of the Lake Pavin Water Column
Source: Front Microbiol. 2022 Jan 10;12:789134. doi: 10.3389/fmicb.2021.789134 (PMC8786505; doi:10.3389/fmicb.2021.789134)
Supplement: Supplementary file 4 [file Data_Sheet_4.docx]

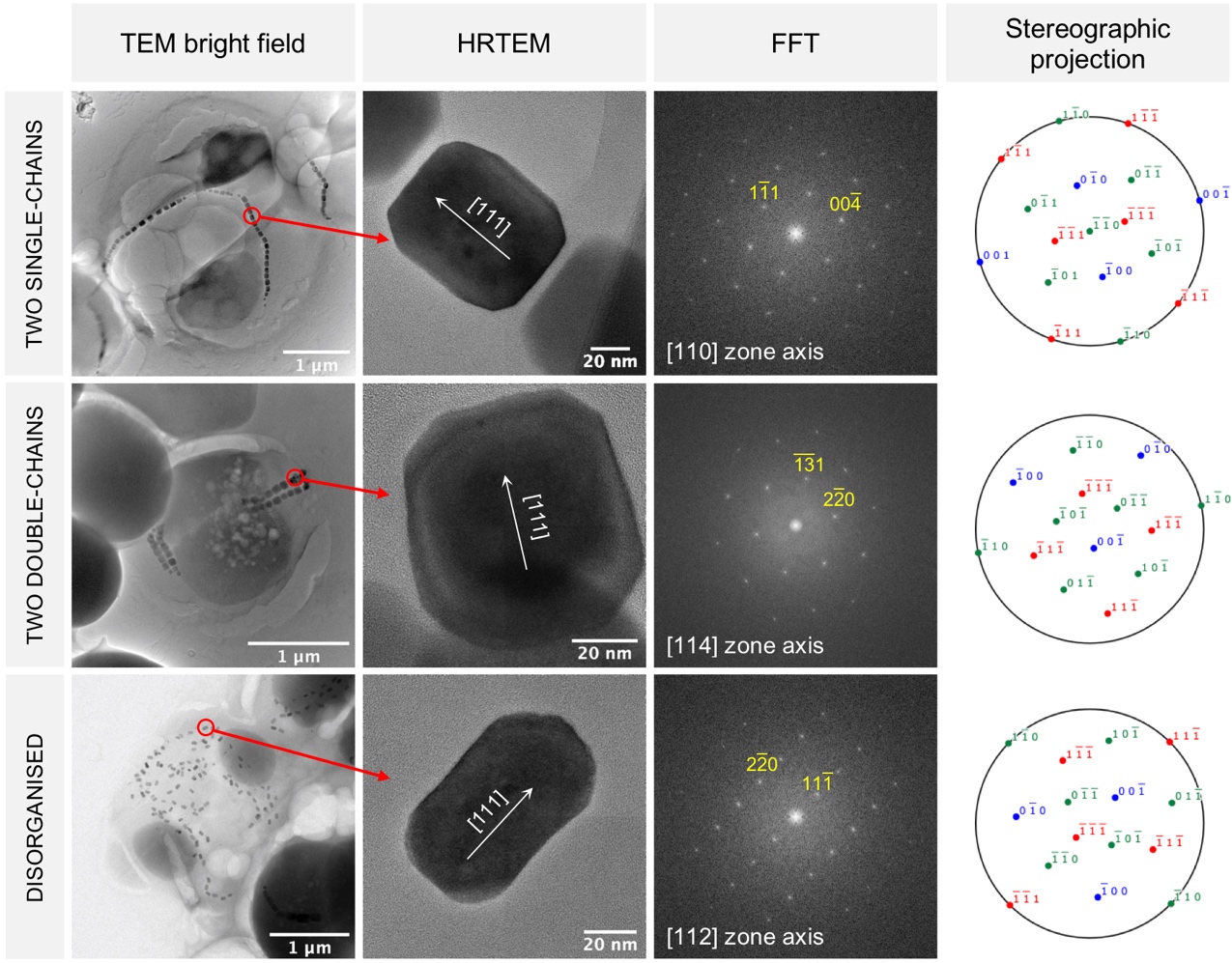


**Supplementary Figure S4.** **Morphological analysis of prismatic magnetosomes in MTBc with three different organisations: two single-chains, two double-chains and disorganised magnetosomes.** The first column contains TEM bright field images of bacteria hosting two single-chains (first row), two double-chains (second row) and disorganised magnetosomes (third row). The second column contains high resolution TEM images (HRTEM) of individual single magnetite crystals for which the corresponding Fast Fourier Transforms (FFT) patterns are shown in the third column. The FFT patterns were calculated using ImageJ software (<https://imagej.nih.gov/ij/>). For each FFT pattern, the observation direction (zone axis) is indicated and some reflections are indexed, providing the exact orientation of each magnetite single crystal. The fourth column presents the stereographic projection oriented with respect to the crystal orientation. From these stereographic projections, it can be deduced that the prismatic magnetosomes are elongated along the <111>. The stereographic projections were computed using SingleCrystal 3.1 software (<http://crystalmaker.com/singlecrystal/>).
