# Supplementary material for: Biogeochemical Niche of Magnetotactic Cocci Capable of Sequestering Large Polyphosphate Inclusions in the Anoxic Layer of the Lake Pavin Water Column
Source: Front Microbiol. 2022 Jan 10;12:789134. doi: 10.3389/fmicb.2021.789134 (PMC8786505; doi:10.3389/fmicb.2021.789134)
Supplement: Supplementary file 5 [file Data_Sheet_5.docx]

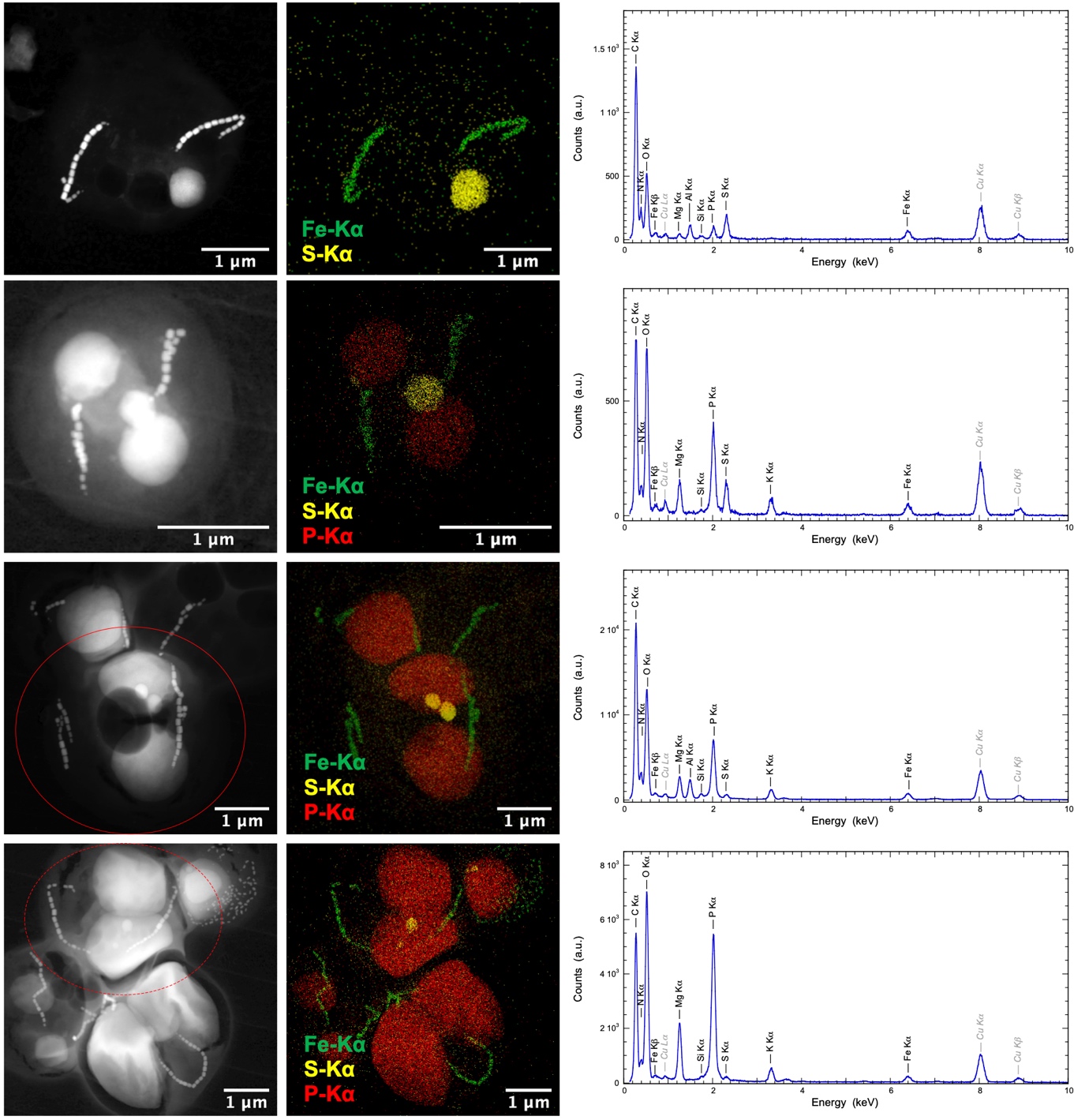


**Supplementary Figure S5.** **Elemental mapping of PolyP and S intracellular inclusions of MTBc with two single-chains magnetosomes.** Each STEM-HAADF images of the cells is correlated to a composite elemental image showing the P, S and Fe elements, and to an X-ray fluorescence spectrum corresponding to the whole composite image. The images show a negative correlation of the sulphur inclusions size over the PolyP inclusions size.
